# Sensitivity of northwest Australian tropical cyclone activity to ITCZ migration since 500 CE

**DOI:** 10.1126/sciadv.add9832

**Published:** 2023-01-11

**Authors:** Rhawn F. Denniston, Caroline C. Ummenhofer, Kerry Emanuel, Roberto Ingrosso, Francesco S. R. Pausata, Alan D. Wanamaker, Matthew S. Lachniet, Kenneth T. Carr, Yemane Asmerom, Victor J. Polyak, Jonathan Nott, Wei Zhang, Gabriele Villarini, John Cugley, Darren Brooks, David Woods, William F. Humphreys

**Affiliations:** ^1^ Department of Geology, Cornell College, Mount Vernon, IA, USA.; ^2^ Department of Physical Oceanography, Woods Hole Oceanographic Institution, Woods Hole, MA, USA.; ^3^ ARC Centre of Excellence for Climate Extremes, University of New South Wales, Sydney, NSW, Australia.; ^4^ Lorenz Center, Massachusetts Institute of Technology, Cambridge, MA, USA.; ^5^ Centres ESCER (Étude et la Simulation du Climat à l’Échelle RÉgionale) and GEOTOP, Department of Earth and Atmospheric Sciences, University of Quebec in Montreal, Montreal, Canada.; ^6^ Department of Geological and Atmospheric Sciences, Iowa State University, Ames, IA, USA.; ^7^ Department of Geoscience, University of Nevada Las Vegas, Las Vegas, NV, USA.; ^8^ Department of Earth, Atmospheric, and Planetary Sciences, Massachusetts Institute of Technology, Cambridge, MA, USA.; ^9^ MIT-WHOI Joint Program in Oceanography/Applied Ocean Science & Engineering, Cambridge and Woods Hole, MA, USA.; ^10^ Department of Earth and Planetary Sciences, University of New Mexico, Albuquerque, NM, USA.; ^11^ College of Science and Engineering, James Cook University, Cairns, QLD, Australia.; ^12^ IIHR-Hydroscience and Engineering, University of Iowa, Iowa City, IA, USA.; ^13^ Department of Plants, Soils and Climate, Utah State University, Logan, UT, USA.; ^14^ Australian Speleological Federation, Perth, WA, Australia.; ^15^ Department of Environment and Science, Queensland Parks and Wildlife Service, Townsville, QLD, Australia.; ^16^ School of Biological Sciences, University of Western Australia, Perth, WA, Australia.; ^17^ Department of Terrestrial Zoology, Western Australian Museum, Welshpool, DC, WA, Australia.

## Abstract

Tropical cyclones (TCs) regularly form in association with the intertropical convergence zone (ITCZ), and thus, its positioning has implications for global TC activity. While the poleward extent of the ITCZ has varied markedly over past centuries, the sensitivity with which TCs responded remains poorly understood from the proxy record, particularly in the Southern Hemisphere. Here, we present a high-resolution, composite stalagmite record of ITCZ migrations over tropical Australia for the past 1500 years. When integrated with a TC reconstruction from the Australian subtropics, this time series, along with downscaled climate model simulations, provides an unprecedented examination of the dependence of subtropical TC activity on meridional shifts in the ITCZ. TCs tracked the ITCZ at multidecadal to centennial scales, with a more southward position enhancing TC-derived rainfall in the subtropics. TCs may play an increasingly important role in Western Australia’s moisture budgets as subtropical aridity increases due to anthropogenic warming.

## Teaser

Stalagmites and climate models reveal ITCZ shifts drove concurrent changes in Australian tropical cyclone and monsoon rainfall.

## INTRODUCTION

Across northern Australia, tropical cyclones (TCs) produce high winds and extreme rainfall that can result in substantial environmental disturbance, socioeconomic disruption, and human fatality (*1*). At the same time, associated high-intensity precipitation and subsequent flooding are important components of moisture budgets and groundwater recharge across the arid Australian subtropics (*2*). Remnants of these storms also provide irregular but substantial rainfall to the agricultural belt in the southwestern portions of the continent (*3*). Recent studies have suggested that the latitudes of TC genesis, maximum intensity, and storm dissipation are shifting poleward, likely in response to anthropogenic influences, but observations of these changes are limited to the most recent few decades (*4*, *5*). While modeling has allowed exploration of the mechanics linking TCs to the intertropical convergence zone (ITCZ) (Supplementary Text) (*6*), reconstructions from proxy records reaching well beyond the instrumental era are necessary to place current changes in a broader context, especially in the more data-sparse Southern Hemisphere.

Most Australian TCs originate in or near the ITCZ (*7*), the highly dynamic component of the tropical atmospheric circulation and center of monsoon rainfall that reflects the meridional energy imbalance across latitudes (*8*). In the Indo-Pacific region, the ITCZ migrates annually from northern Australia to southern Asia, tracking differential summer heating of the oceans and continents, and is argued to have shifted its maximum latitude in the summer hemisphere in response to, among other factors, aerosols (*9*–*11*), solar irradiance (*12*), and Arctic ice cover (*13*). The sensitivity of TC activity to changes in ITCZ location is difficult to evaluate using the short observational record. Factors such as sea surface temperature also influence cyclogenesis, steering, and storm strength, and thus, it cannot be assumed that rainfall derived from TCs responds simply and uniformly to shifts in ITCZ position. This issue may be amplified during intervals with mean climate conditions distinct from the modern era [e.g., the Little Ice Age (~1450–1850 CE) and the Medieval Climate Anomaly (~850–1250 CE)] (*14*). One additional approach to exploring this relationship lies in the use of climate model simulations, but these are generally not highly skilled at capturing positioning of the ITCZ over Australia (*15*) and have not yielded close agreement on projections of TC activity (*16*). Alternatively, proxy-based TC reconstructions can be compared to positioning of the ITCZ over previous eras with distinct climate states. For example, multicentennial periods of elevated TC frequency from the Caribbean correlate with a more southerly ITCZ position (*17*). However, such studies have been limited by a lack of proximal, high-resolution reconstructions of the ITCZ (*18*–*22*); are often located at sites experiencing long storm recurrence intervals; and, to our knowledge, have focused exclusively on the Northern Hemisphere.

**Fig. 1.**
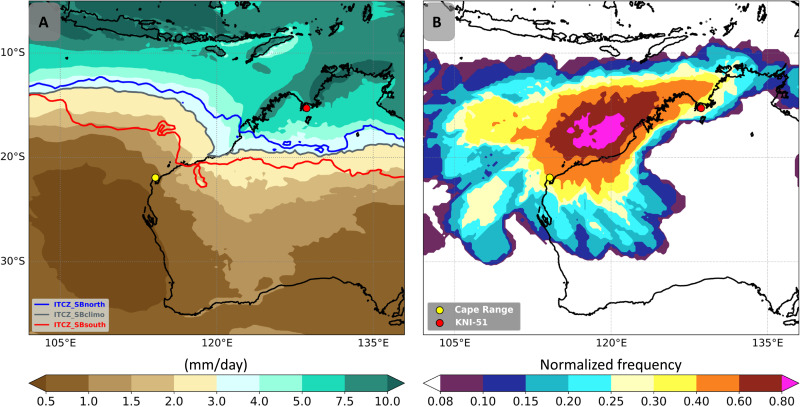
Australian tropical wet season and TC climatology. (**A**) Mean tropical wet season (December to March) rainfall calculated with ERA5 data for the period 1979–2018 for the Australian region with the southern boundary (SB) of the austral summer ITCZ (defined as the isohyet at 3 mm/day) for the mean (gray), southward (red), and northward (blue) positions (Materials and Methods). Circles denote the locations of the cave sites (KNI-51, red; Cape Range, yellow). (**B**) Normalized TC frequency (number of TCs passing over each grid cell divided by the maximum value over the entire map) calculated with ERA5 data (spatial grid at 0.25° horizontal resolution) for the Australian region cyclone season (November to April) for 1979–2018 CE (Materials and Methods).

In this study, we explore the relationship between ITCZ migration and TC activity at an unprecedented temporal scale by leveraging highly resolved, hydroclimate-sensitive stalagmite records from the tropics and subtropics of Australia. This represents an unusual circumstance, wherein one stalagmite record preserves evidence of TC activity (*23*) and the other (this record) preserves evidence of monsoon variability, with both reflecting positioning of the ITCZ over northwestern Australia. We couple these records with a cutting-edge downscaling of TCs in a climate model simulation of the last millennium. Here, we present a composite stalagmite record from the tropical Australian cave, KNI-51 (15.3°S, 128.6°E, ~100 m above sea level) (red circle in Fig. 1; fig. S1). Oxygen isotopes preserve monsoon rainfall variability, which reflects meridional shifts in the ITCZ. We integrate this time series with an existing annually resolved, stalagmite-based reconstruction of TC activity from Cape Range, subtropical Western Australia (yellow circle in Fig. 1) to provide a unique test of the influence of ITCZ migration on TC activity. These records span most of the past 1500 years and thus offer a robust test of the nature of ITCZ/TC interactions during periods with marked shifts in ITCZ position. The results hold important implications for our understanding of TC dynamics and their impacts on moisture budgets across the semiarid and drought-prone subtropics through time.

## RESULTS

### Western Australia ITCZ and TC reconstructions

Cave KNI-51 is located near the northern margin of the austral summer ITCZ, making rainfall at this site highly sensitive to shifts in its position. Deeper penetration of the ITCZ into the continental interior is associated with enhanced rainfall across the Western Australia tropics (fig. S2). Annual precipitation at KNI-51 averages ~850 mm, ~80% of which is contributed by the Australian summer monsoon between December and March (fig. S3). TCs track near the cave in some but not all years and provide 10 to 20% of average annual total rainfall (*24*, *25*). Monsoon strength is mostly reflected in oxygen isotopes of rainfall through the so-called “amount effect,” i.e., the intensity of precipitation at a given location owing to changes in the strength of convection and atmospheric humidity (*26*, *27*). The isotopic signal of tropical rainfall, particularly in near-coastal settings and including in TCs, involves the depletion of ^18^O owing to a complex interplay of evaporation and recycling of water droplets during descent from clouds, Rayleigh distillation, the depth of convection, and microcloud dynamics (*28*, *29*). Together, these mechanisms drive an inverse relationship between the amount of rainfall and oxygen isotope ratios (^18^O/^16^O) of rainfall reaching the land surface. The amount effect is robust at KNI-51 using weighted monthly mean values [Pearson’s correlation coefficient (*r*) = 0.66; *P* < 0.0001] and weighted annual means (fig. S4, Materials and Methods, and data S1). We also interpret the oxygen isotope data as integrated rainfall at and upwind of the cave location, such that the rainwater oxygen isotope values are lower when regional rainfall is higher, as demonstrated at Darwin (*r* = 0.70; *P* < 0.05), located 400 km to the northeast (*27*).

**Fig. 2.**
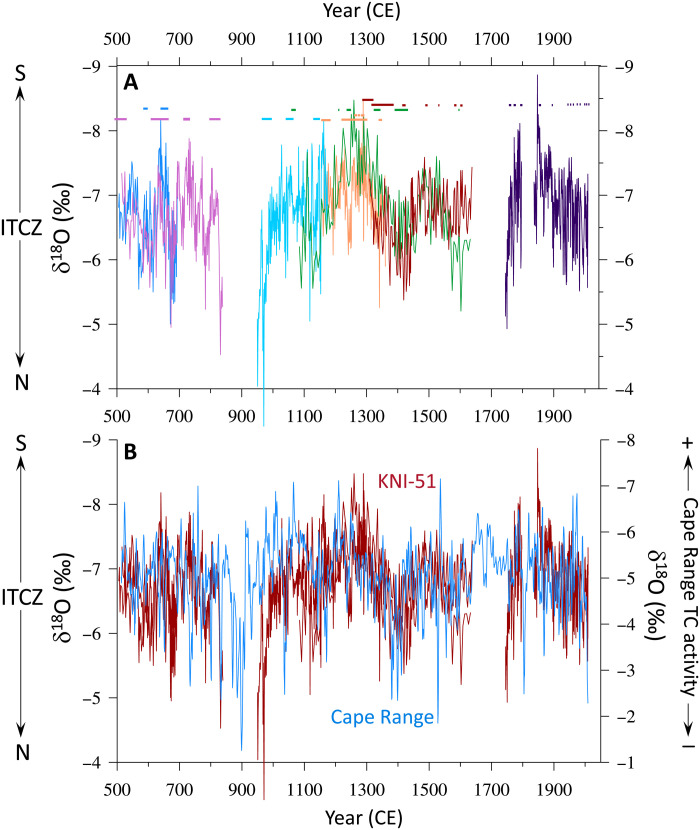
Western Australia ITCZ and TC reconstructions. (**A**) KNI-51 stalagmite oxygen isotope record. Each stalagmite is shown in a different color (with horizontal bars representing 2 SD error windows on corresponding U/Th dates). More negative oxygen isotope values correspond via the amount effect to increased monsoon rainfall driven by a southward shift in ITCZ position. (**B**) Stacked KNI-51 record (red; Materials and Methods) and Cape Range stalagmite oxygen isotope record (blue). Increased contributions of TC-derived rainfall lower the oxygen isotope values at Cape Range.

The KNI-51 ITCZ reconstruction is derived from nine aragonite stalagmites, four of which were previously published (fig. S5 and Materials and Methods) (*9*, *30*). The high growth rates (1 to 2 mm/year) and high U abundances allow for precise chronological control and, thus, an unprecedented look into monsoon variability of the Australian tropics over the past 1500 years. Each stable isotope sample integrates less than 1 year of growth. Several stalagmites partially overlap in age, and the oxygen isotope ratios among coeval samples share similar values and trends, demonstrating that secondary effects (e.g., evaporation and kinetics) have not diluted the primacy of the climate signal (Fig. 2A, Materials and Methods, and fig. S6). The high number of stalagmites comprising this time series allows for rigorous tests of replication, and two stalagmites were excluded from the composite KNI-51 record based on offsets in δ^18^O values (fig. S6 and Materials and Methods). Growth models are derived from 56 high-precision U/Th dates (24 newly presented here), with most characterized by 2 SD age uncertainties ranging between ±1 and 30 years (Materials and Methods, fig. S7, and table S1); all ages fall in stratigraphic order. The only gaps in the record span 840–950, 1640–1750, and 1800–1835 CE (Fig. 2A). Environmental conditions in KNI-51 (Materials and Methods and fig. S8), the absence of secondary calcite, consistency of radiometric dates, and coherence of stalagmite δ^18^O data convincingly argue that stalagmite oxygen isotope variability reflects shifts in the oxygen isotopic of precipitation; thus, we interpret the KNI-51 record as preserving evidence of changes in monsoon rainfall over time (*31*, *32*).

TC activity from the Western Australia subtropics is reconstructed using a published stalagmite record from Cape Range (22.0°S, 114.0°E) (*23*). This time series is derived from oxygen isotopic ratios sampled at annual resolution from 1500 stalagmite growth laminae (ending at 2010 CE and used to establish the chronology) and uses the fact that TC rainfall is generally depleted in ^18^O beyond ambient precipitation (*33*). The Cape Range stalagmite δ^18^O record does not contain a statistically significant relationship with seasonal or annual rainfall totals or the number of rain days, and interannual isotopic variability is far in excess of what can be explained by changes in cave temperature. Oxygen isotope values of individual lamina were tested against historical records of TC activity over two decades (1990–2010 CE) using TC observations that explain 40% of the stalagmite δ^18^O variance (Materials and Methods and fig. S9).

In contrast to KNI-51, Cape Range is located well poleward of the ITCZ (650 km south of KNI-51 and ~300 km south of today’s ITCZ southern boundary; Fig. 1A). The average annual rainfall of 230 mm is only a quarter of that received at KNI-51 and is distributed more evenly throughout the year, with the wettest months generally receiving only 30 to 40 mm (Fig. 1A and fig. S3A). Cape Range is also situated just southwest of the region in Australia with the highest average number of TCs per year, with most TCs tracking southwest (Fig. 1B) (*34*). Along the northwest Australian coast just east of this zone of peak TC activity, TCs contribute more than 50% of 1-, 2-, and 3-day annual maximum rainfall totals, exceeding 50 mm, and this percentage decreases to the south and north (fig. S10) (*35*). Thus, mean rainwater (and also cave drip water) is highly dependent on the genesis location and steering of TCs. Moreover, it is likely that the Cape Range stalagmite record disproportionately reflects large precipitation events associated with TCs because other sources of rainfall (e.g., northwest cloud bands) (*36*) generally yield smaller rain totals, which, in the region’s warm and dry climate, minimize their impact on groundwater recharge. In contrast, the KNI-51 stalagmite record is biased against extreme rainfall events, as these regularly trigger short-term (fig. S8, B and C) cave flooding that submerges stalagmites and temporarily interrupts stalagmite crystallization (*37*). Thus, Cape Range’s more arid climate and the recurring contribution by TCs to effective moisture make interannual precipitation variability markedly larger than in the Kimberley region (fig. S11).

## DISCUSSION

### Covariance of the ITCZ and Western Australia TCs

Comparison of the KNI-51 ITCZ and Cape Range TC reconstructions reveals notable similarities, including at multidecadal and centennial scales, for the last eight centuries (1170–2009 CE; *r* = 0.49; *P* = 0.02 for data in 30-year bins; Materials and Methods). The strength of the correlation diminishes to 0.37 (*P* = 0.05) for the last millennium and falls further for periods before 850 CE (Materials and Methods). Several phenomena may account for this, including unidentified hiatuses and/or missing or false annual laminae that could have interfered with the band counting chronology (*38*) or nonstationarity in the climate system.

Two possible mechanisms, both involving shifts in the austral summer ITCZ, can explain the covariance of the tropical (KNI-51) and subtropical (Cape Range) stalagmite oxygen isotope time series. First, meridional changes in the ITCZ could have altered the delivery of tropical (monsoon-related) rainfall to Cape Range as occurs at KNI-51. We consider it unlikely that this explanation accounts for most of the observed correlation. First, while rainwater associated with non-TC tropical systems can be more ^18^O-depleted than mean precipitation at Cape Range, these appear to provide relatively small amounts of moisture to groundwater recharge or cave drip water (fig. S12 and Materials and Methods). Thus, a northward migration of the ITCZ would only slightly reduce tropical moisture to Cape Range and would have a negligible impact on oxygen isotope ratios; the area would be sensitive predominantly to a southward shift in the ITCZ that increased the contribution of tropical moisture to regional water budgets.

**Fig. 3.**
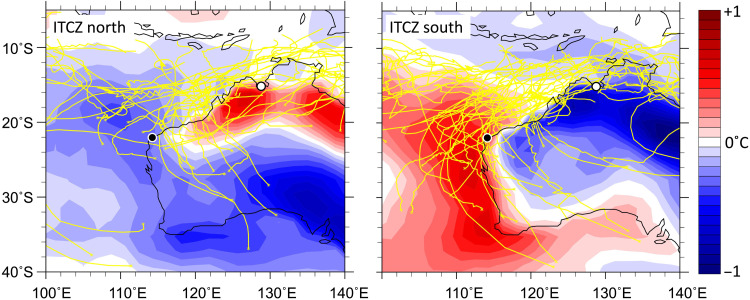
Western Australia TC tracks associated with ITCZ position. Australian TC tracks (yellow lines; data from IBTrACS) (*41*) and TC season (November to April) skin temperature anomalies based on NCEP/NCAR reanalysis data for the period 1979–2016 for years when the southern boundary of the ITCZ is positioned 1 SD or more north (left) or south (right) of the mean. Cave sites denoted by circles: (KNI-51, white; Cape Range, black).

Alternatively, shifts in ITCZ position could have affected the genesis latitude, steering, or intensity of TCs along the northwest Australian shelf. As previously mentioned, TCs frequently provide large amounts of rainfall to Cape Range, and precipitation from these storms is typically characterized by very ^18^O-depleted signatures. Marsh sediments proximal to Cape Range also provide evidence of distinct, multicentennial rainfall regimes over the last two millennia, including one characterized by enhanced aridity punctuated with large, episodic floods during portions of the Little Ice Age, when the ITCZ appears to have been located more equatorward (*39*). To test the potential link between TCs and the ITCZ, we used the ERA5 reanalysis dataset (*40*) to calculate which years between 1979 and 2018 CE contained an anomalously (more than 1 SD from the mean) northward- or southward-positioned ITCZ (based on the southern boundary of the ITCZ; Materials and Methods) and then examined TC tracks for those years (Figs. 1A and 3 and table S2) (*41*). This analysis reveals that TCs at Cape Range are consistent with changes in ITCZ position, with both the total number of TCs along the northwest shelf of Australia and the percentage of storms passing close (<200 km) to Cape Range increasing when the ITCZ is displaced further south (Fig. 3 and fig. S13).

However, the limited number of years available for observational analysis, coupled with the irregular and infrequent occurrence of TCs in any given year, complicates our ability to develop statistically significant results. In addition, the percentage of annual rainfall ascribed to TCs is not differentiated between an anomalously northward or southward austral summer ITCZ. Thus, to better investigate the origins of this relationship, we turn to a dynamical-statistical downscaling that allows us to simulate large numbers of synthetic TCs using ERA5 reanalysis as driving data (Materials and Methods) (*42*, *43*). TC tracks are created by randomly seeding, in space and time, the evolving global, large-scale environment. For each of 40 years (1979–2019 CE), we generated 150 events (6150 events total) passing within 200 km of Cape Range using conditions set by ERA5. Well over 99% of the seeded tracks dissipated rapidly and were discarded; the survivors constitute the downscaled TC climatology of the original reanalysis or climate model.

**Fig. 4.**
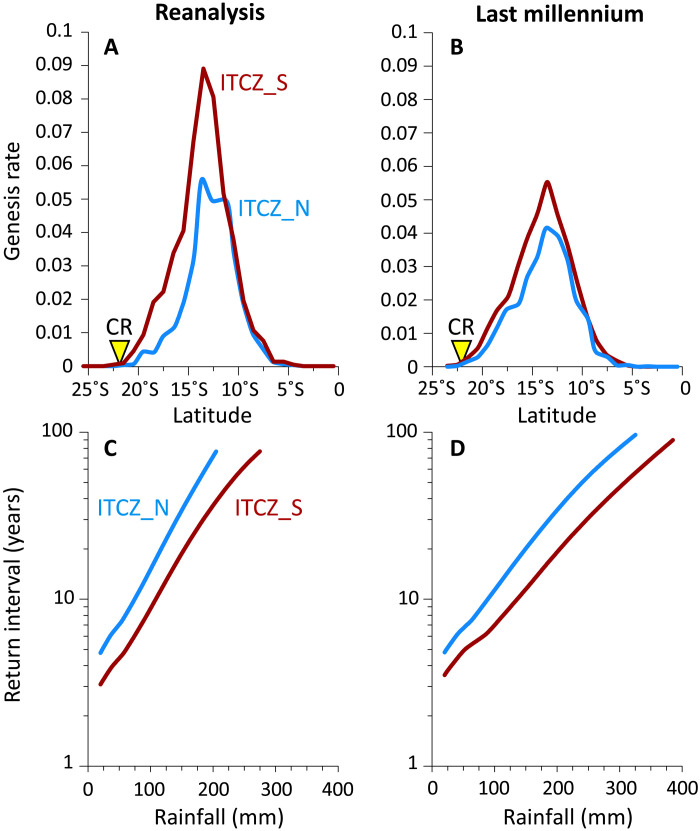
Results of downscaled TC seeding model experiments. Change in TC genesis rate with latitude for northwest Australia for the northerly (red) and southerly (blue) position of the ITCZ southern boundary based on data from ERA5 reanalysis for all years from 1979 to 2018 (**A**) and Max Planck Institute Earth System Model (MPI-ESM) “Past-1000 Year” simulation (**B**). Comparison of frequency and rainfall of TCs affecting Cape Range for southerly and northerly ITCZ southern boundary based on data from ERA5 reanalysis (**C**) and the MPI-ESM simulation (**D**). CR, Cape Range.

This downscaling technique reveals that a southward shift in the ITCZ southern boundary increases storm tracks at Cape Range by elevating the TC genesis rate just equatorward of this site (Fig. 4A), a finding supported by the observational TC track dataset (International Best Track Archive for Climate Stewardship, IBTrACS) (Fig. 3) (*41*). Moreover, an increase in TC rainfall over Cape Range is detected; for storms with return periods of less than 100 years, TC rainfall is ~30% higher than in years with a northerly ITCZ (Fig. 4C). Near Cape Range, increases in storm intensity are also enhanced in the model during southerly ITCZ years, suggesting that the reduction in Cape Range stalagmite oxygen isotope ratios reflects more frequent development of stronger storms that increase the contribution of ^18^O-depleted rain to cave drip water (figs. S10 and S11).

Interannual variability in the numbers of Australian TCs has been previously investigated and linked predominantly to a variety of drivers, including the El Niño–Southern Oscillation (ENSO), Indian Ocean Dipole (IOD), Southern Annular Mode (SAM), Madden-Julian Oscillation, and Interdecadal Pacific Oscillation (*44*, *45*). Of these, ENSO is generally considered to represent the most prominent control on TC activity, and so, we investigated its possible impact on our findings. Across northwest Australia, La Niña years generally experience elevated numbers of TCs, while El Niño years see reduced numbers of TCs, owing to changes in environmental factors such as vorticity and midlevel humidity (*46*, *47*). Given that ENSO also influences monsoon and pre-monsoon season rainfall across many parts of northern Australia, it is therefore possible that the covariance between the KNI-51 and Cape Range records reflects changes in the phase (El Niño versus La Niña), spatial pattern (e.g., central versus eastern Pacific ENSO), frequency, or strength of ENSO events. As a test of this hypothesis, we categorized the northerly and southerly ITCZ years according to ENSO state. Over the 40 years of ERA5 data used in this analysis, El Niño and La Niña events occur in both northerly and southerly ITCZ years. Of the 7 years with a northerly ITCZ, 2 years were La Niña, 2 years were El Niño, and 3 years were neutral, while for those with a southerly ITCZ, 5 years were La Niña and 2 years were El Niño (including the very strong 1982 event) (table S2). Additional evidence that the covariance of the KNI-51 and Cape Range records is not modulated by ENSO alone involves its impact on monsoon rainfall at KNI-51. While substantial reductions in rainfall are associated with El Niño events across many parts of Australia, the impact of ENSO on rainfall in the Western Australia tropics is muted (*5*). These observations are consistent with the findings of a 39-year study that found northwest Australian TCs to be less dependent on ENSO than many other basins around the globe, including the rest of the Australian region (*48*). Together, it appears that the observed covariance between the Cape Range and KNI-51 records does not strictly reflect an ENSO signal unless the variance of these events was markedly stronger over the last two millennia than today, a question that is made more difficult to answer by the uncertainty surrounding ENSO dynamics over this time (*49*). The roles played by other important drivers of Australian TC activity, such as the IOD and SAM, also seem to lack a coherent relationship with ITCZ position (table S2), although their variability before the instrumental period is also poorly constrained (*50*).

**Fig. 5.**
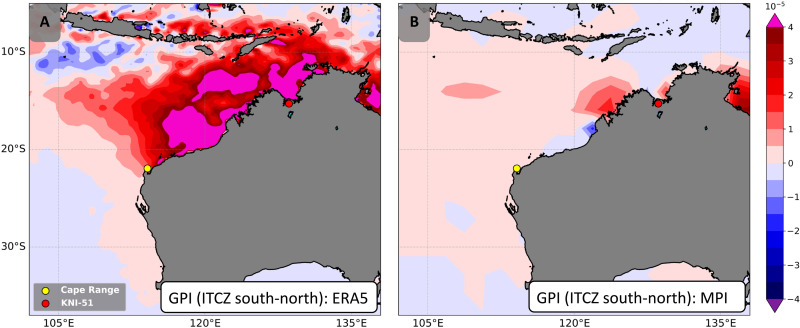
TC genesis potential index (GPI) associated with ITCZ position. Differences in TC GPI for southerly-northerly ITCZ based on ERA5 data for November to April, 1979–2018 CE (**A**) and on output from the MPI-ESM (**B**). Statistical significance is shown in fig. S17. Circles denote cave sites: KNI-51, red; Cape Range, yellow.

Given the brevity of the ERA5 dataset relative to the recurrence interval of TCs at Cape Range and our desire to test the ITCZ/TC relationship during periods with climate states distinct from the modern, we applied this TC downscaling approach to a model simulation of the last millennium (850–1849 CE) performed using the Max Planck Institute Earth System Model (MPI-ESM) (*51*). To minimize the impact of ENSO in this portion of the analysis, we used only years characterized as ENSO-neutral based on tropical Pacific Ocean surface water temperatures (Materials and Methods). As with the ERA5 analysis, the mean position of the ITCZ southern boundary was calculated, and downscaling was performed on years when the ITCZ southern boundary was positioned at least 1 SD north or south of the mean. Consistent with reanalysis data for the last 40 years, downscaling the MPI-ESM for the last millennium reveals an increase in TC genesis rates and rainfall at Cape Range associated with a more southerly ITCZ (Fig. 4). Driving these differences are a reduction in vertical wind shear and middle troposphere saturation deficit, and an increase in potential intensity (figs. S14 and S15), which, when integrated, comprise the TC genesis potential index (GPI) (Fig. 5 and Materials and Methods) (*52*); changes in relative vorticity were small (fig. S16). The difference between TC rainfall between a northerly and southerly ITCZ location is somewhat reduced in the model relative to the reanalysis data, a fact that may be attributable to the coarser resolution of the model relative to the reanalysis (fig. S17). Positioning of the ITCZ over Australia is an issue with other climate models of the last millennium (e.g., CMIP5) (*15*), but the location of the ITCZ south of the center of modern TC activity aligns quite well with the region of highest track density downscaled from the MPI-ESM (fig. S18). These concurrent poleward shifts support the pronounced sensitivity of TCs to positioning of the ITCZ across northwest Australia as inferred from the KNI-51 and Cape Range stalagmite records (Fig. 2).

### Implications for extratropical moisture budgets

Australia is one of the most drought-sensitive regions on Earth, and climate simulations project increasing moisture stress in coming decades due to rising temperatures and changes in the atmospheric tropical overturning circulation (*53*, *54*), the so-called “widening of the tropics” (*55*). In most semiarid climates such as subtropical Western Australia, large precipitation events such as those associated with TCs are primarily responsible for aquifer recharge and basin filling (*56*). This includes Lake Carnegie, a 5700-km^2^ basin located at the border of the Gibson and Little Sandy Deserts, which is occasionally filled to overflowing by TCs (bom.gov.au; fig. S18). TCs also provide important rainfall to middle latitude agricultural zones (*57*), such as when TC Seroja made landfall north of Perth in 2021, delivering up to 150 mm to the Wheatbelt of southwest Australia (fig. S18). Such large precipitation events recharge aquifers, raise water levels in rivers and basins, increase deep soil moisture, and reduce fire risk. Thus, the close coupling of TCs and the ITCZ demonstrated here holds important ramifications for water budgets, agricultural activity, and fire projections.

Climate simulations suggest that rising greenhouse gas concentrations will trigger changes to factors influencing cyclogenesis (e.g., vertical wind shear and relative vorticity) in the Australian region, such that the number of TCs will be reduced in coming decades (*58*, *59*). Models also suggest that little change in ITCZ location will occur in response to rising atmospheric carbon dioxide levels, although an equatorial shift has been projected for the western Pacific during the monsoon/TC season in Australia (*60*). While not expected to migrate substantially over Western Australia, positioning of the ITCZ in monsoonal areas is complex and dependent on regional boundary conditions (*61*), and rainfall from TCs is likely to intensify as the surface ocean warms (*62*). Monsoon variability recorded by stalagmites at KNI-51 and cave sites across southern China reveal that the ITCZ migrated poleward and equatorward synchronously at multidecadal to centennial scales in both hemispheres over recent millennia (*9*). One possible consequence of this expansion/contraction behavior may be that TC activity across South Asia changed concomitantly with the shifts identified for northern Australia. Thus, anticipating future contributions of TCs to (sub)tropical moisture budgets in both hemispheres requires developing a detailed dynamical framework, including both position and width, for the Indo-Pacific ITCZ (*59*, *60*).

## MATERIALS AND METHODS

### Cave setting, environmental monitoring, and stable isotope measurements

KNI-51 is entered through its single small entrance, and all stalagmites were collected from a single chamber located approximately 500 m from the entrance. KNI-51 stalagmites are cylindrical and range in height from 230 to 1100 mm. To minimize impacts on the cave environment, we collected only stalagmites that were already broken and down (except one: KNI-51-11). Cave conditions (i.e., air temperature, barometric pressure, relative humidity, and water level) were measured in sub-hourly intervals for a 3-year span using HOBO U23 and U20L data loggers. Temperatures during the austral tropical wet season (December to March), when stalagmite growth occurs at KNI-51 (drips are observed to cease in the stalagmite chamber by June), experience only small to moderate (±0.3°C) intra- or interseasonal variability (fig. S7).

Each stalagmite was bisected using a water-cooled trim saw and sampled using a handheld Dremel tool. Stable isotope ratio measurements were performed on the previously unpublished KNI-51 stalagmites at Iowa State University using a Thermo Finnigan Delta Plus XL mass spectrometer. The average analytical uncertainty based on isotopic standards (NBS-19 and NBS-18) was ±0.11‰ (per mil) 1σ. Replication of oxygen isotope values and trends in coeval samples represents a robust method of identifying disequilibrium crystallization (*9*). Replication between overlapping KNI-51 stalagmites at multidecadal scales is robust in all but two cases: KNI-51-16-7 (1325–1650 CE) and KNI-51-10 (a previously published sample), with the data in these stalagmites higher than coeval samples by 0.7 and 1.2‰, respectively (fig. S6). The origins of these discrepancies at KNI-51 are unknown; although in China (*63*) and southeastern Australia (*64*), they have been attributed to preferential evaporation of water in the epikarst and the frequency of recharge events. As a result of these offsets, stalagmites KNI-51-10 and KNI-51-16-7 were excluded from the integrated KNI-51 δ^18^O time series.

Rainwater samples analyzed for stable isotopes were collected each morning from October 2015 to February 2020 by volunteers in Kununurra, ~50 km south of the cave, using a Productive Alternatives rain gauge. Rainfall amounts at the cave site were obtained from the Western Australia Department of Water and Environmental Regulation weather station at Eight Mile Hill, located 5 km from the cave (Rimco tipping bucket positioned 1 m above the ground). Oxygen and hydrogen isotopic ratios in rainwater were measured by cavity ring-down laser spectroscopy using a Picarro L2130-i isotopic liquid water analyzer. All stable isotope values are reported in per mil VPDB (VSMOW). For water, at least one reference standard (VSMOW, IAEA-OH-2, or IAEA-OH-3) was analyzed after every fifth sample and used for regression-based isotopic corrections, with an average analytical uncertainty for oxygen of ±0.10‰ 1 SD.

The widespread application of stalagmites as paleoenvironmental proxies is based, in part, on their resistance to diagenetic alteration. KNI-51 stalagmites are composed of aragonite, a more soluble polymorph of calcite, and thus are susceptible to recrystallization, leaching, or open-system behavior (*65*, *66*). No calcite is apparent in any of these stalagmites [calcite KNI-51 stalagmites reported in (*30*) are not included in this study, owing to markedly slower growth rates and considerably larger age uncertainties]. As convincingly argued in (*67*), it is exceedingly unlikely that similar isotopic values and trends of coeval stalagmites from the same or nearby caves could originate if one or both of the stalagmites had experienced secondary alteration. The data presented here for KNI-51 are perhaps the most robustly replicated of any published late Holocene stalagmite time series.

The Cape Range stalagmite oxygen isotope record was converted to a TC reconstruction using a modern calibration of TC proximity. Their resulting index of TC activity is defined as the average accumulated energy expended over the TC season within a given proximity to Cape Range and was centered on detrended stalagmite (and thus precipitation) δ^18^O values of recently deposited stalagmite laminae. Corrections for tropical moisture were made in (*23*) using the stalagmite oxygen isotope record from southern Indonesia in (*68*). So as not to introduce model-derived uncertainty into our comparison of the Cape Range and KNI-51 datasets, we use the raw Cape Range stalagmite data rather than these cyclone index values (fig. S9).

### Age models

U/Th dates of stalagmite aragonite were performed using a Neptune multicollector inductively coupled plasma mass spectrometer at the University of New Mexico. Unsupported ^230^Th was corrected using an initial ^230^Th/^232^Th ratio of 4.4 parts per million (±100%), but because of high U and low ^232^Th abundances, corrected ages are not particularly sensitive to the initial Th ratio. Age models were constructed using COPRA age modeling software (*69*). The age model of a previously published stalagmite time series (KNI-51-10) (*30*) was adjusted through the introduction of a hiatus (unrecognizable by visual inspection) that improves the fit of the growth model to the radiometric dates. However, this stalagmite was excluded from the composite dataset, owing to offsets in δ^18^O (fig. S6).

### Correlation statistics

Discrete stalagmite oxygen isotope ratios were integrated into a single time series based on COPRA-derived model ages for each individual stable isotopic analysis. Pearson correlation coefficients and *P* values between the KNI-51 and Cape Range records were calculated on oxygen isotope values averaged within nonoverlapping 30-year bins. The size of this time interval was chosen to approximate age model uncertainties. KNI-51 data from individual stalagmites were integrated (stacked) by age before being binned based on the COPRA model ages. Assembling the data into 30-year bins mitigates issues associated with age uncertainties on discrete data points. Correlations are strongest over the last 800 years, possibly suggesting the presence of unidentified hiatuses in the Cape Range record. Correlation and probability values for different intervals are as follows: 1185–2009 CE (*r* = 0.49, *P* = 0.015), 1095–2009 CE (*r* = 0.44, *P* < 0.02), 825–2009 CE (*r* = 0.24, *P* = 0.18), and 525–2009 CE (*r* = 0.12, *P* = 0.43). Correlations could also be modestly enhanced by tuning the KNI-51 time series to the Cape Range record using error windows on the KNI-51 chronology.

### ITCZ, TC, and moisture source analysis

We use the high-resolution (25 km) ERA5 reanalysis (*40*) for the period 1979–2018 to perform a set of composite analyses in which we compare the austral summer precipitation and TC activity during periods of anomalous southward and northward shifts of the austral summer ITCZ. The ITCZ is defined here as the mean of the local maximum of precipitation. The southern boundary of the zone of major tropical convection is defined as the location where the climatological austral summer (December-February) rainfall reaches 3 mm/day using the approach in (*70*). These authors noted a shift in the position of the austral spring ITCZ between 90° and 110°E, northwest of the zone of peak TC activity in northwestern Australia, with some years characterized by pronounced (up to 1000 km from the mean) equatorward shifts in ITCZ position. Unlike over the Indian and Pacific oceans where the ITCZ is well defined, the ITCZ over the Maritime Continent is relatively diffuse owing to the region’s land-sea distribution and complex topography and the deep convection resulting from the high sea surface temperatures of the Indo-Pacific Warm Pool. In this area, a clear and zonally consistent ITCZ is unlikely (*7*), but smaller-scale convergence zones would likely respond similarly to large-scale external forcing that drive positioning of the ITCZ.

In our analysis, the years in which the southern boundary of the ITCZ is located more than 1 SD further north (south) of its climatological average are considered as those with anomalous northward (southward) shifts. On the basis of these data, we evaluated the differences between TC-derived rainfall for ITCZ-north and ITCZ-south years at Cape Range and found that they were not statistically distinct using both a nonparametric test (Mann-Whitney test, *P* value above 5% significance level) and the bootstrap method (*71*) with 100 randomly selected 7-year samples of the 40-year (1979–2018) distributions of TC-derived seasonal differences (below 89% confidence level). It is unclear whether this is simply a function of the vagaries of individual storms and the abbreviated nature of the observational record. We similarly evaluated periods of La Niña with El Niño, wherein El Niño (La Niña) years were identified as those in which the December to February NINO3.4 index was above (below) +0.4°C (−0.4°C). In the MPI-ESM, ENSO modes were determined from air surface temperature. The ENSO index is a 5-month running mean of the weighted mean temperature anomalies calculated over the NINO3.4 area, where anomalies are between the single year and its 200-year climatology. ENSO-neutral years occur when index values are between −0.4° and 0.4°C. This approach identified a total of 777 ENSO-neutral years between 850 and 1850 CE, 45 of which were ITCZ-north and 164 were ITCZ-south.

For TCs, we use the TC tracking algorithm developed in (*72*). A three-step procedure (storm identification, storm tracking, and storm lifetime) is used to detect TCs, as is also done in previous studies (*73*, *74*). However, this algorithm also performs a double-filtering approach similar to that applied in (*75*) to ensure that the genesis and dissipation phases of TCs are well represented and that TCs are not counted twice in case of a temporary decrease intensity followed by a restrengthening. For more details, please refer to (*72*). TC rainfall amounts were calculated for days when the eye of a TC was located within 500 km of the site.

To investigate the influence of tropical moisture not derived from TCs, the source of moisture associated with precipitation events at Cape Range was calculated for the years 1979–2018 CE by applying a moisture tracking model to the ERA5 reanalysis data [see (*76*) for model details]. The dates during which TCs were located within a 500-km radius of Cape Range were identified using the Australian Bureau of Meteorology TC tracking protocol (bom.gov.au/cyclone/tropical-cyclone-knowledge-centre/history/tracks) and excluded from the dataset (fig. S11).

The GPI is calculated as

$$\mathrm{GPI}\equiv {|\eta |}^{3}\chi^{-4/3}\mathrm{MAX}{\left[(V_{\mathrm{pot}}-35\;{\mathrm{ms}}^{-1}),0\right]}^{2}\times {\left(25\;{\mathrm{ms}}^{-1}+V_{\mathrm{shear}}\right)}^{-4}$$

where η is absolute vorticity of the 850-hPa flow, *V*_pot_ is the potential intensity, *V*_shear_ is the magnitude of the 850- to 250-hPa wind shear, and

$$\chi \equiv \frac{S_{b}-S_{m}}{S_{0}^{*}-S_{b}}$$

where *S*_b_, *S*_m_, and S^*^_0_ are the moist entropies of the boundary layer and middle troposphere and the saturation moist entropy of the sea surface, respectively. The quantity χ is a nondimensional measure of the saturation deficit of the middle troposphere but is used here in the inverse of its traditional form such that χ becomes lower as the middle troposphere becomes drier (figs. S14 and S15) (*52*). The statistical method (Wilcoxon test) used to evaluate these differences would be more robust if it included a larger number of years; we plot the *P* values in fig. S17.

### Downscaled TC modeling

For the present study, we use the downscaling method developed in (*42*, *43*). TC tracks are created by randomly seeding, in space and time, the evolving, global, large-scale environment, and these events were then transformed into Southern Hemisphere TC convention, where the year begins on July 1 and ends on June 30. This environment is synthetically generated from gridded global reanalyses or climate models in a way that ensures that the monthly means of all variables are those of the gridded data (interpolated to the storm positions) and that the monthly mean variances and covariances of the daily atmospheric winds with respect to their monthly means are correct. Last, the kinetic energy spectrum of the synthesized large-scale winds obeys geostrophic turbulence scaling.

Once the tracks are created, the Coupled Hurricane Intensity Prediction System (CHIPS) (*77*) model is run along each of the randomly generated tracks. The intensity model has very high spatial resolution in the storm core, owing to the use of an angular momentum radial coordinate, and has been shown to produce skillful real-time intensity forecasts (*77*). Well over 99% of the seeded tracks dissipate rapidly and are discarded; the survivors constitute the downscaled TC climatology of the original reanalysis or climate model. This technique has been shown to accurately simulate all the salient features of the current climatology of TCs when applied to global reanalysis data (*43*).

There are several advantages to this technique in comparison to conventional downscaling using regional models. The use of angular momentum coordinates allows increasing spatial resolution of the storm core as its intensity increases, such that each storm’s intensity is limited by the physical properties of its environment rather than by numerical resolution. Because the TC model is driven by the statistics of the global model or reanalysis, an arbitrarily large number of events can be simulated in a given climate, and the seeding is global so there is no need to preselect subdomains. Owing to the considerably larger dataset (850–1849 CE), each year in the MPI-ESM was seeded with only 50 events, rather than the 150 used with the reanalysis data.

## Supplementary Materials

### This PDF file includes:

Supplementary Text Figs. S1 to S18 Tables S1 and S2 References
