# Supplementary material for: Sensitivity of northwest Australian tropical cyclone activity to ITCZ migration since 500 CE
Source: Sci Adv. 2023 Jan 11;9(2):eadd9832. doi: 10.1126/sciadv.add9832 (PMC9833654; doi:10.1126/sciadv.add9832)
Supplement: Supplementary file 1 — Supplementary Text Figs. S1 to S18 Tables S1 and S2 References [file sciadv.add9832_sm.pdf]

Science Advances
AAAS

Supplementary Materials for

Sensitivity of northwest Australian tropical cyclone activity to ITCZ migration since 500 CE

Rhawn F. Denniston *et al.*

Corresponding author: Rhawn F. Denniston, rdenniston@cornellcollege.edu

Sci. Adv. **9**, eadd9832 (2023)
DOI: 10.1126/sciadv.add9832

The PDF file includes:

Supplementary Text
Figs. S1 to S18
Tables S1 and S2
Legend for data S1
References

Other Supplementary Material for this manuscript includes the following:

Data S1

Supplementary Text

ITCZ Positioning and Impacts on TCs

Positioning of the ITCZ reflects the energy balance across latitudes (*8*) and summer heating of land masses pull the ITCZ poleward relative to adjacent oceans (*78*). In one high resolution study of global TC frequency, shifts in the position of the ITCZ were found to be partly responsible for impacting cyclogenesis (*79*).

The frequency of tropical cyclones has been linked to the degree of poleward displacement of the ITCZ. For example, ref. 6 demonstrated that in an aqua-planet with a slab ocean, an imposed cross-equatorial oceanic heat flux shifts the ITCZ poleward, resulting in a global increase in TC frequency. Likewise, ref. 80 showed that volcanically induced ITCZ shifts change the distribution of TC genesis and other metrics, favoring the hemisphere in which the ITCZ resides.

Supplementary Figures

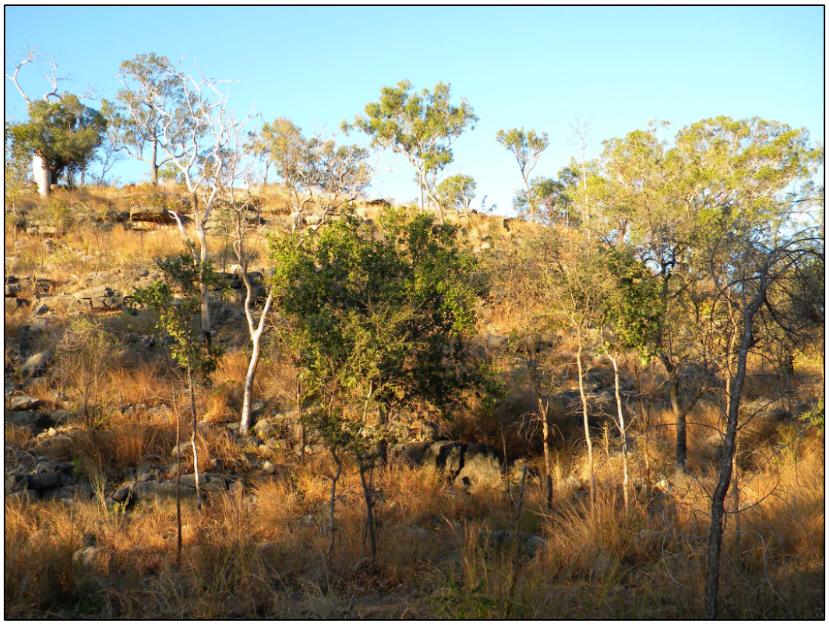

Figure S1. Landscape near KNI-51. Low-lying hills of the eastern Kimberley, Western Australia. Devonian limestone is regularly exposed through the area's thin soils. Vegetation is predominantly Terminalia spp. and boab trees between understory grasses. Photo credit: R. Denniston.

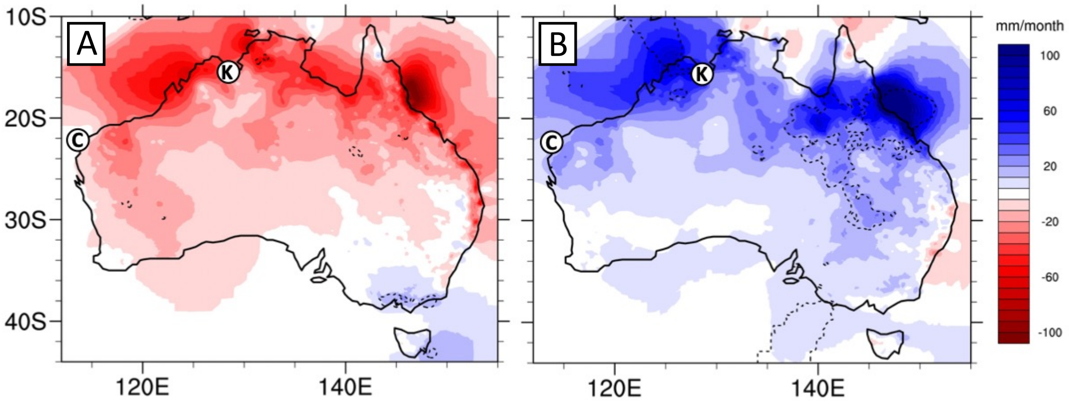

Figure S2. Australian December-March rainfall anomalies associated with northerly (A) and southerly (B) ITCZ. Circles denote locations of KNI-51 (K) and Cape Range (C). Time period is 1979-2018 based on AGCD (*81*). Anomalies encircled by stippled lines denote statistical significance at the 95% confidence level relative to mean December-March conditions over the same time period.

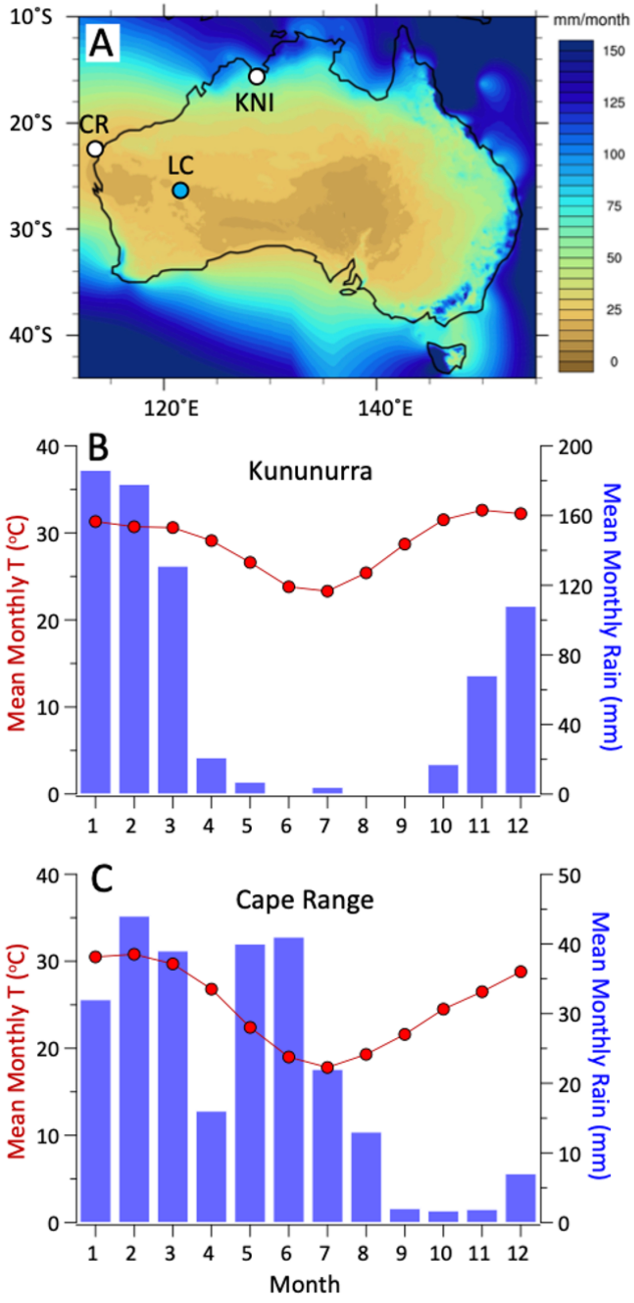

Figure S3. Australian cave site climatologies. A. Monthly average annual precipitation for the hydrologic year May-April from 1950-2018. Data from AGCD (*81*). KNI = KNI-51. CR = Cape Range. LC = Lake Carnegie. **B.** Mean monthly rainfall and temperature from Kununurra, located in the eastern Kimberley near KNI-51 (1986-2018). Data from Kununurra airport weather station (*BOM.gov.au*). **C.** Mean monthly rainfall and temperature Cape Range. Data from Learmonth airport weather station (*BOM.gov.au*).

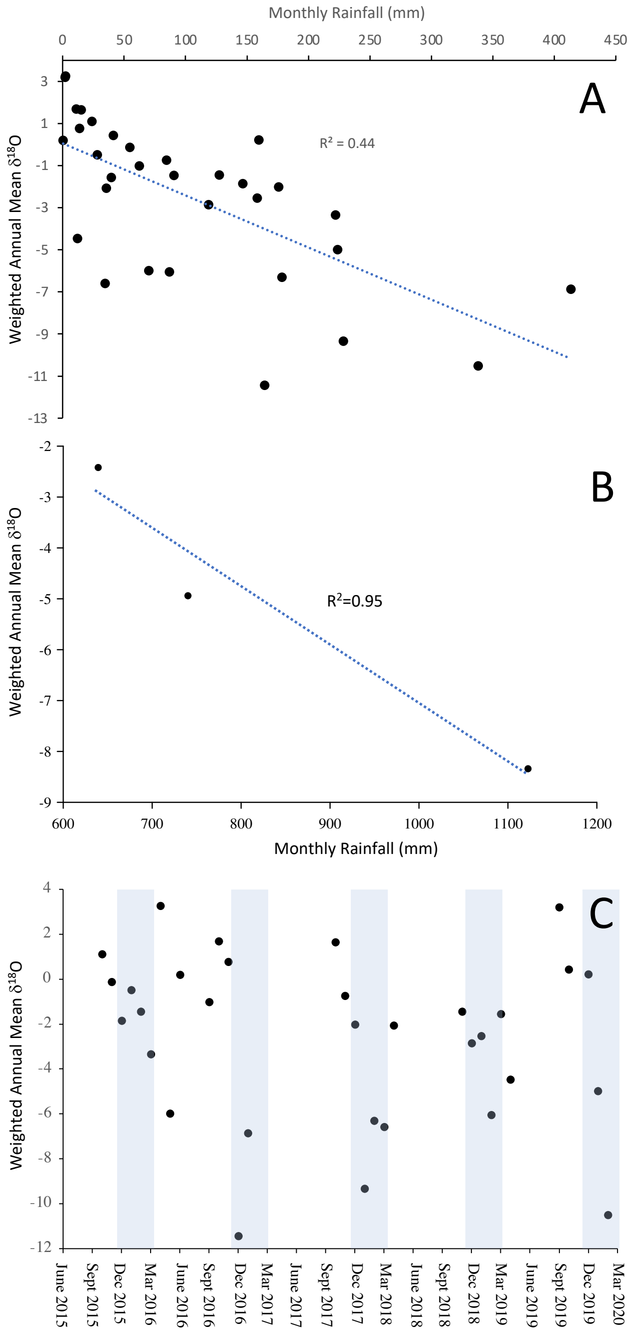

Figure S4. Amount effect at KNI-51. A. Weighted mean monthly oxygen isotope ratios of precipitation collected at Kununurra for June 2015 - February 2020 versus monthly rainfall. Owing to the strong seasonality of rainfall in tropical Western Australia, many austral winter months contained too little precipitation to be sampled. **B.** Same as (a) but for weighted mean annual values. **c.** Same as (a) but plotted by month. Blue bars denote wet season (DJFM). Sampling technique is discussed in the Methods section of manuscript. For data see Supplemental Data file.

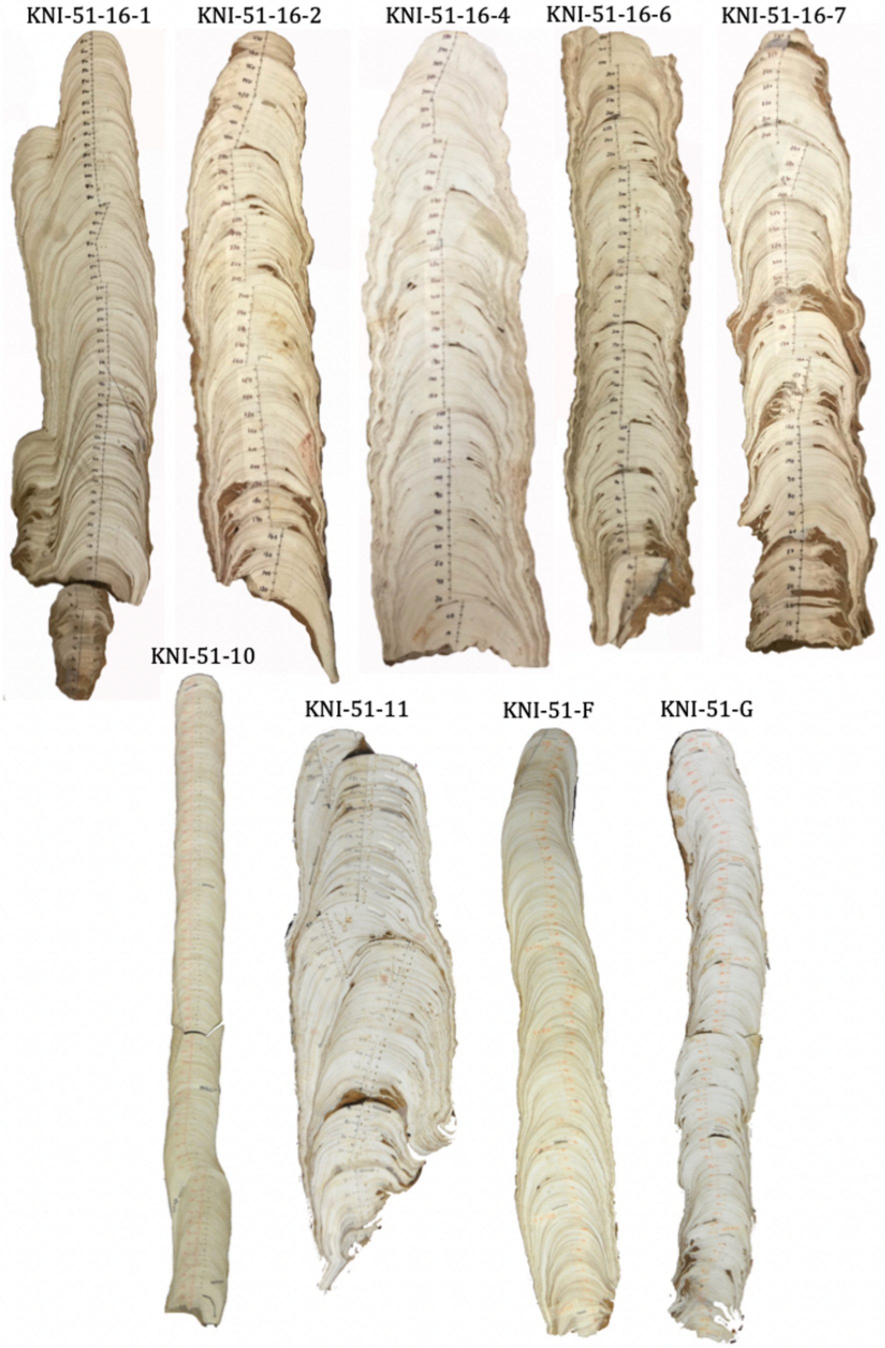

Figure S5. KNI-51 stalagmites used in this study. Dark brown sections parallel to growth surfaces are sedimentary layers deposited when stalagmite caps are submerged during cave flood events. Stalagmites are composed of microcrystalline aragonite and show no signs of secondary alteration in their petrography or stable isotope or U-series geochemistry[82]. KNI-51-10 and KNI-51-16-7 were excluded from the composite $\delta^{18}O$ times series owing to offsets in $\delta^{18}O$ values. Numbers along central growth axis represent mm from the stalagmite base. Stalagmites KNI-51-10, -11, -F, and -G were previously published.

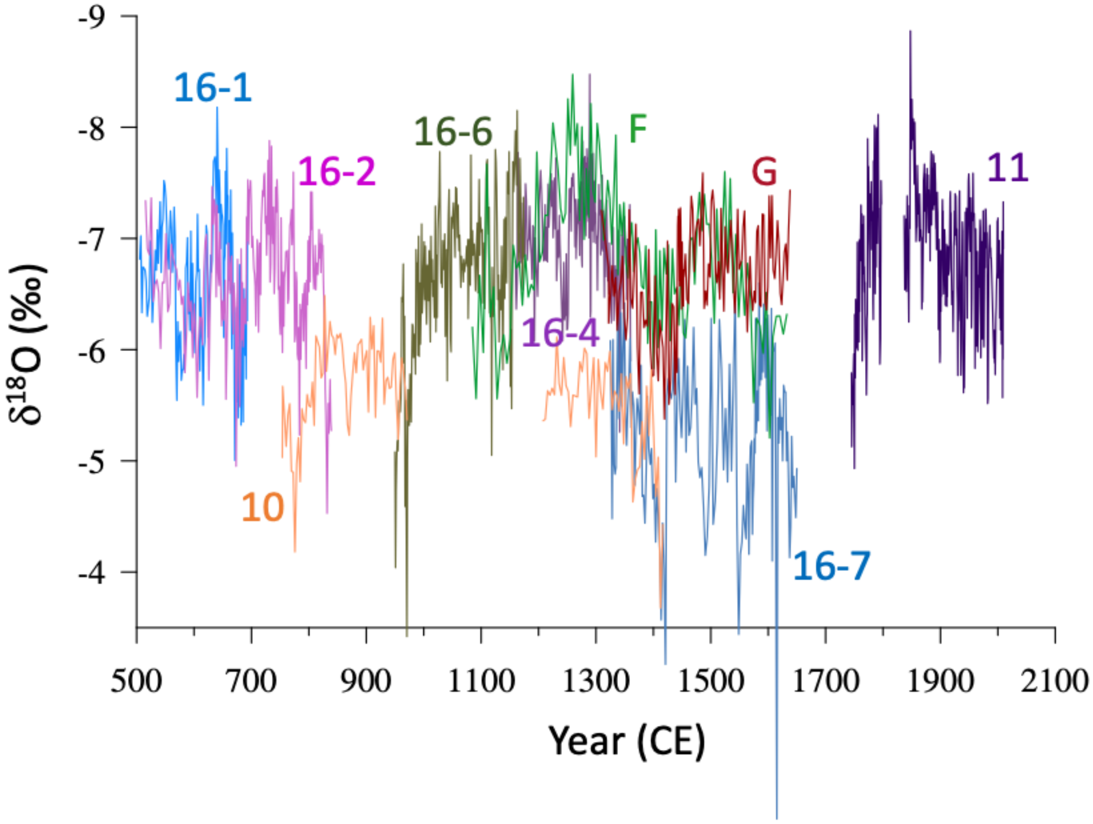

Figure S6. All KNI-51 stalagmite isotope values. Same as Figure 2A (each stalagmite shown in a different color) but including the two stalagmites (KNI-51-10 and KNI-51-16-7) that are offset in $\delta^{18}O$. Discrepancies in oxygen isotopic composition between coeval stalagmites can be related to the dripwater itself (e.g., distinct levels of evaporative enrichment of ^{18}O in the epikarst; mixing of multiple reservoirs (distinct ages of fluids) of infiltrated water), or may arise during crystallization (i.e., different degrees of kinetic fractionation between dripwater and aragonite at the stalagmite growth horizon) (*31-32, 63-64*).

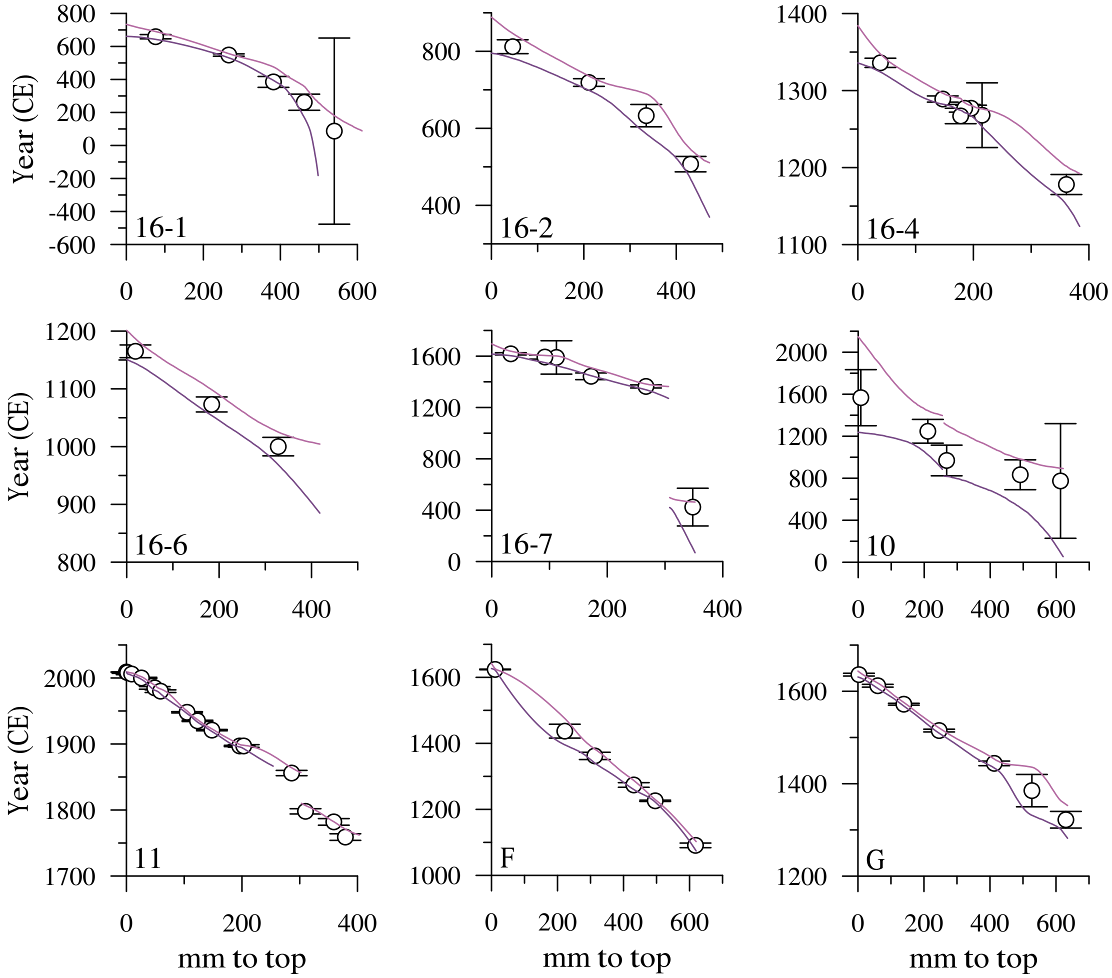

Figure S7. KNI-51 stalagmite age models. U-Th ages (circles) with 2 s.d. errors. Lines define the 2.5% and 97.5% confidence window. Derived using COPRA age modeling software (*69*). Stalagmites KNI-51-16-1, 16-2, 16-4, 16-6, 16-7 are newly presented here. The age model for stalagmite KNI-51-10 has been modified from its originally published version through the addition of a hiatus (Methods).

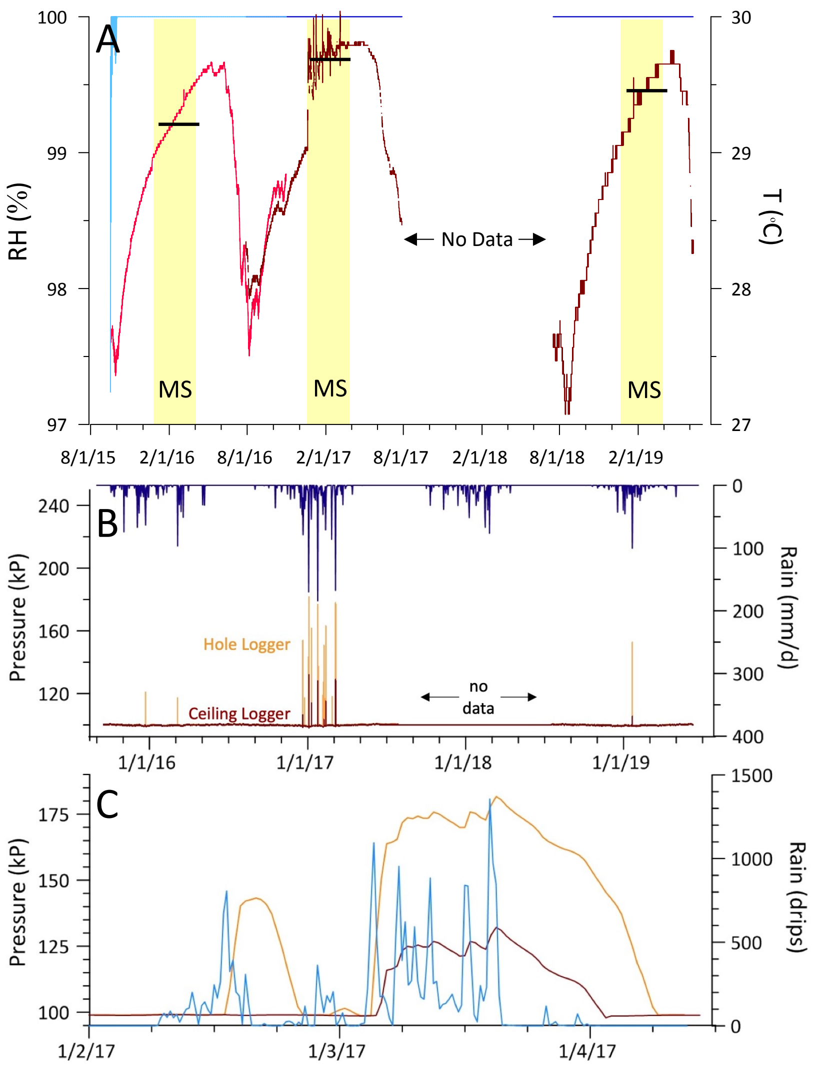

Figure S8. KNI-51 cave monitoring. A. Relative humidity (RH) and air temperature in the stalagmite chamber. Yellow bars denote monsoon season (MS; December-March), with black horizontal lines defining mean temperatures during these intervals. **B.** Rainfall (blue) at KNI-51 (from Eight Mile Mill rain gauge; Methods) compared to water levels (denoted by spikes in pressure due to water standing over the logger) in the KNI-51 stalagmite chamber. The "hole logger" was installed at the lowest point in the chamber, ~2 m below the mean cave floor elevation, while the "ceiling logger" was installed 0.5 m below the roof of the chamber. Note the regular flooding of the cave during large rain events. **C.** Water level measurements during a 3-day period in January 2017 for the two water level data loggers. Rainfall measurements (blue) were taken above the cave using a Pluvimate automated rainwater sampler. Data are shown as drip rate rather than mm/hour. Note the short duration (typically <24 hours) of cave flooding events. The majority of stalagmites were broken, down, and buried within mud on the cave floor when collected, and this sediment layer precluded alteration of aragonite.

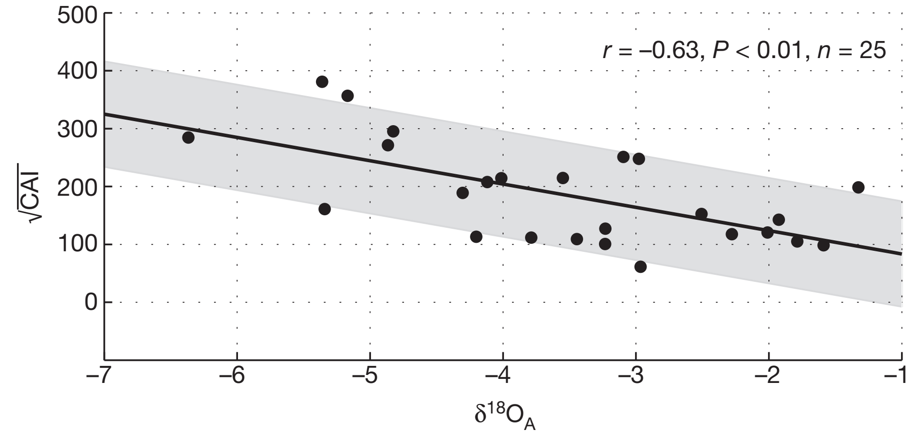

Figure S9. Development of the Cape Range stalagmite TC reconstruction. This figure from ref. 23 denotes the calibration of a site-specific TC reconstruction method derived from oxygen isotope values measured in actively growing, annually laminated stalagmites. Each TC that passed within 400 km of Cape Range (as well as a separate cave site in northeast Australia) was assessed along its path according to duration, distance to the cave site, maximum wind speed, and radius of maximum wind speed over the course of the calibration period (CE 1990-2010). A composite value reflecting these effects was calculated for each year of the calibration period and compared with the oxygen isotope value of the stalagmite lamina for the corresponding year. A Cyclone Activity Index (CAI) was then constructed that reflects the average accumulated energy expended over a given TC season within range of the cave site. This includes the lifespan, size, and intensity of each TC as well as its distance from the site at each point in time. Unlike other TC indexes such as the accumulated cyclone energy index (*83*) or the power dissipation index (*52*), the CAI is focused on TC rainwater oxygen isotope values and targets a given location (i.e., the cave). Grey shaded area denotes root mean squared error of the difference between actual and modeled values. Reproduced with permission.

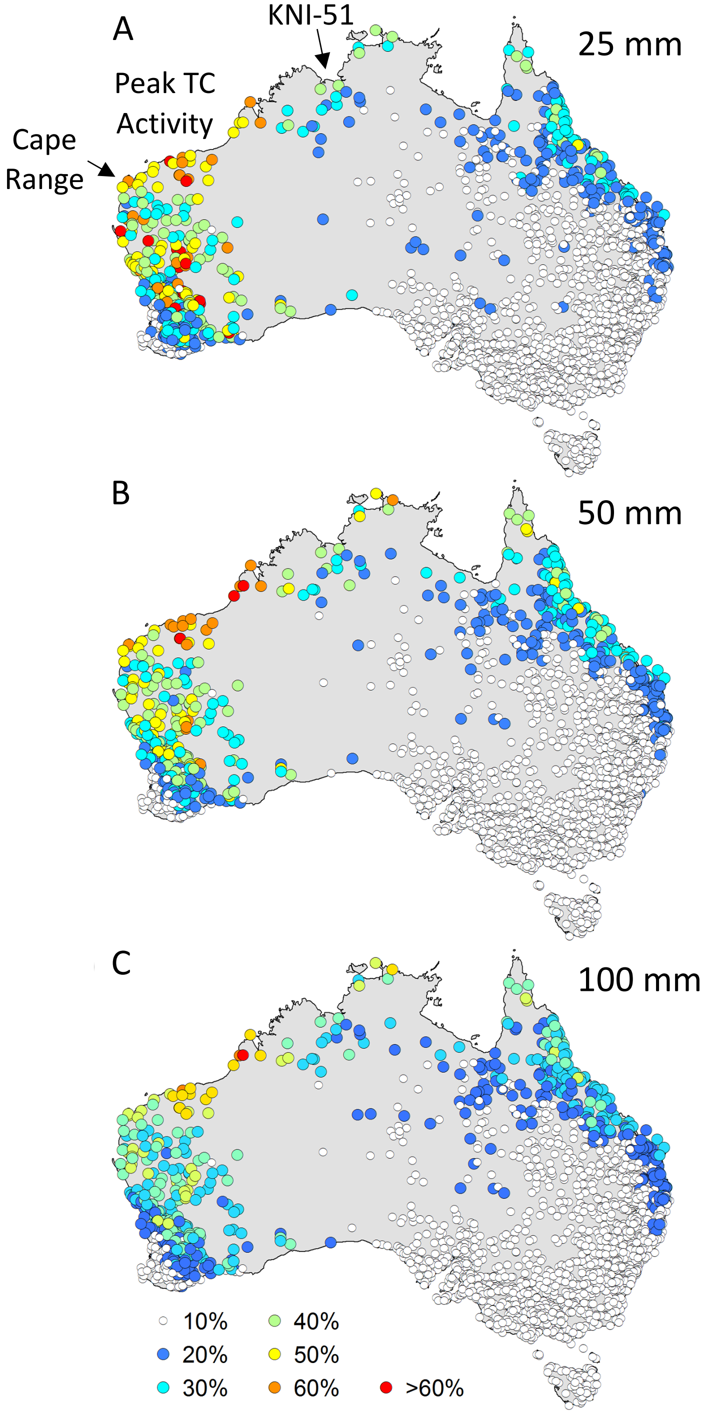

Figure S10. Contributions of TCs to 2-day Australian annual maximum daily rainfall. Values exceeding (A) 25 mm, (B) 50 mm, (C) 100 mm. The contribution of TCs decreases with distance northeast and south of zone of peak TC activity, which lies between Cape Range (CR) and cave KNI-51. After ref. 35. Reproduced with permission.

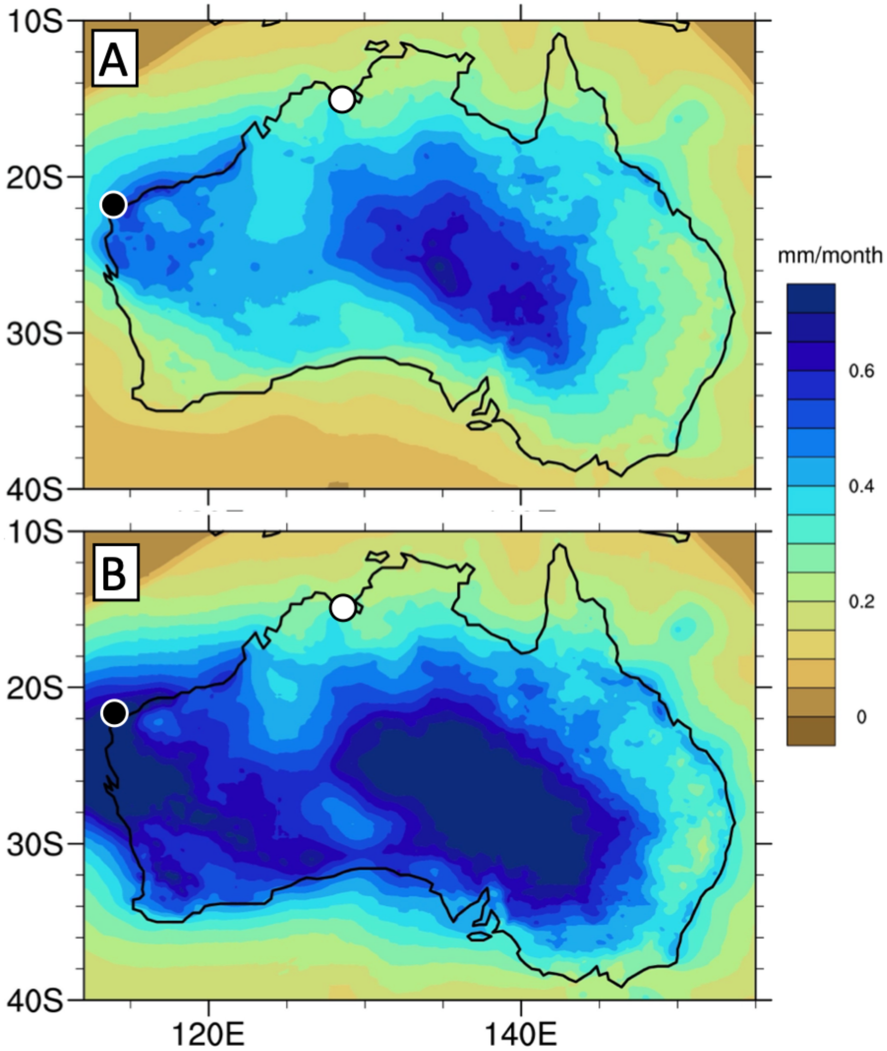

Figure S11. Coefficient of variation in precipitation for Australia (CE 1950-2020). A. Australian tropical wet season of December-April. **B.** Hydrologic year of May-April. Cave sites denoted by circles: KNI-51 = white; Cape Range = black. Calculated using data from AGCD (*81*). Note enhanced variability at Cape Range for both time intervals.

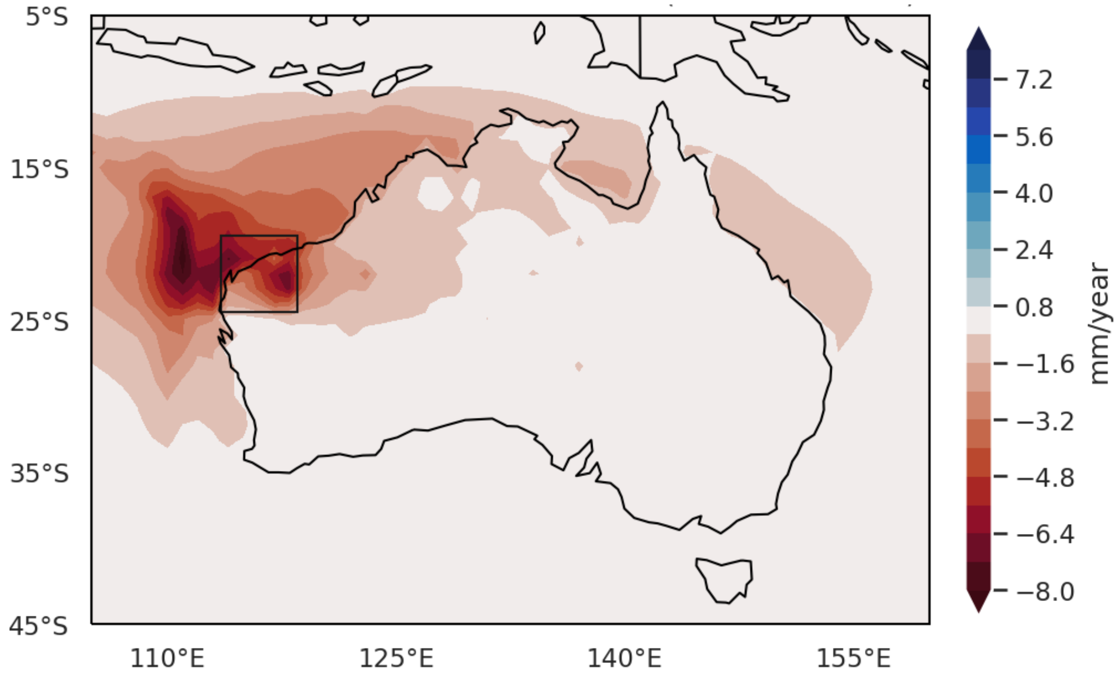

Figure S12. Moisture sources for Cape Range. Absolute difference in moisture contribution to precipitation in the Cape Range area (rectangle) precipitation by source region between TC and non-TC days (TC-days minus non-TC days; January 1979-December 2018; units of mm per year). TC-days are defined as days when the eye of a TC is within 500 km of Cape Range. TC tracks were determined using the Australian Bureau of Meteorology tropical cyclone website (http://www.bom.gov.au/cyclone/tropical-cyclone-knowledge-centre/history/tracks/). Negative values (shown in red) correspond to regions from which non-TC events contribute more moisture than TC events. Though non-TC events contribute a majority of total moisture to Cape Range precipitation, the majority of this moisture is derived from sources proximal to Cape Range and thus non-TC events from the deep tropics are not expected to meaningfully contribute to cave drip water oxygen isotope values.

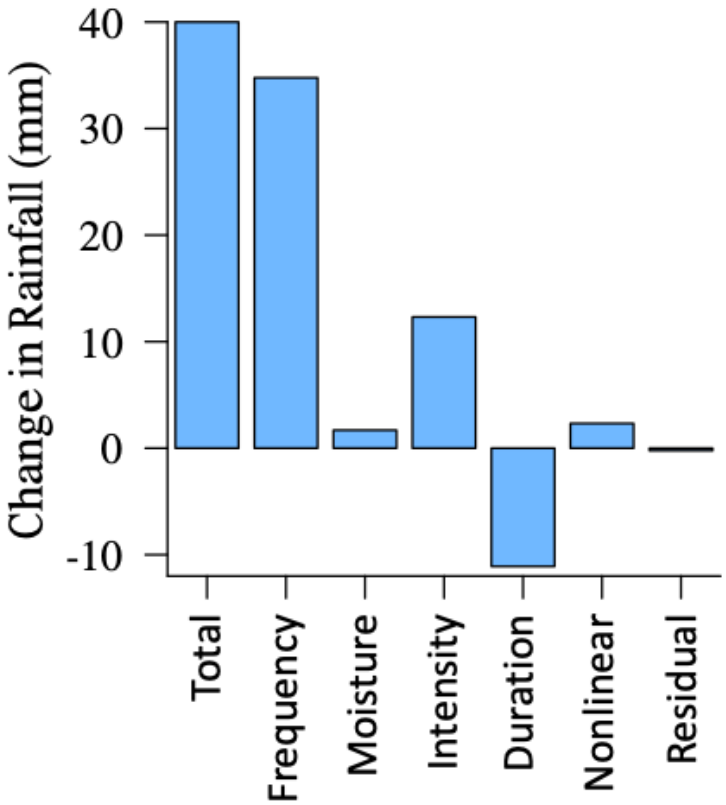

Figure S13. Attribution of TC rainfall at Cape Range from downscaling. Results of downscaled climate model simulations based on ERA5 data for 20-year return interval TCs at Cape Range. Total rainfall anomaly (left-most bar) is explained by changes in storm frequency, moisture, intensity, duration, and non-linearities.

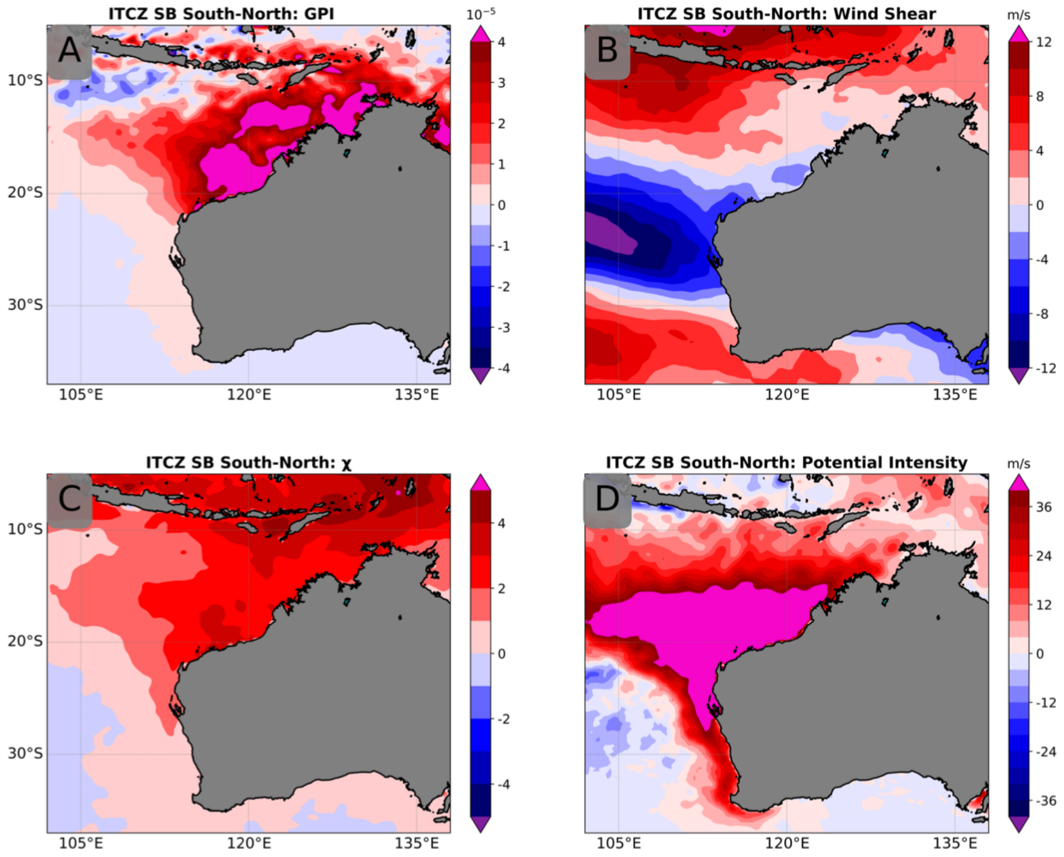

Figure S14. Genesis potential index (GPI) and associated controls on cyclogenesis. Data from ERA5. (A) Genesis potential index; (B) vertical wind shear; (C) χ is a nondimensional measure of the saturation deficit of the middle troposphere; here we use the inverse of the traditional definition such that χ is proportional rather than inversely proportional to relative humidity (*52*); (D) potential intensity (see Methods for more information).

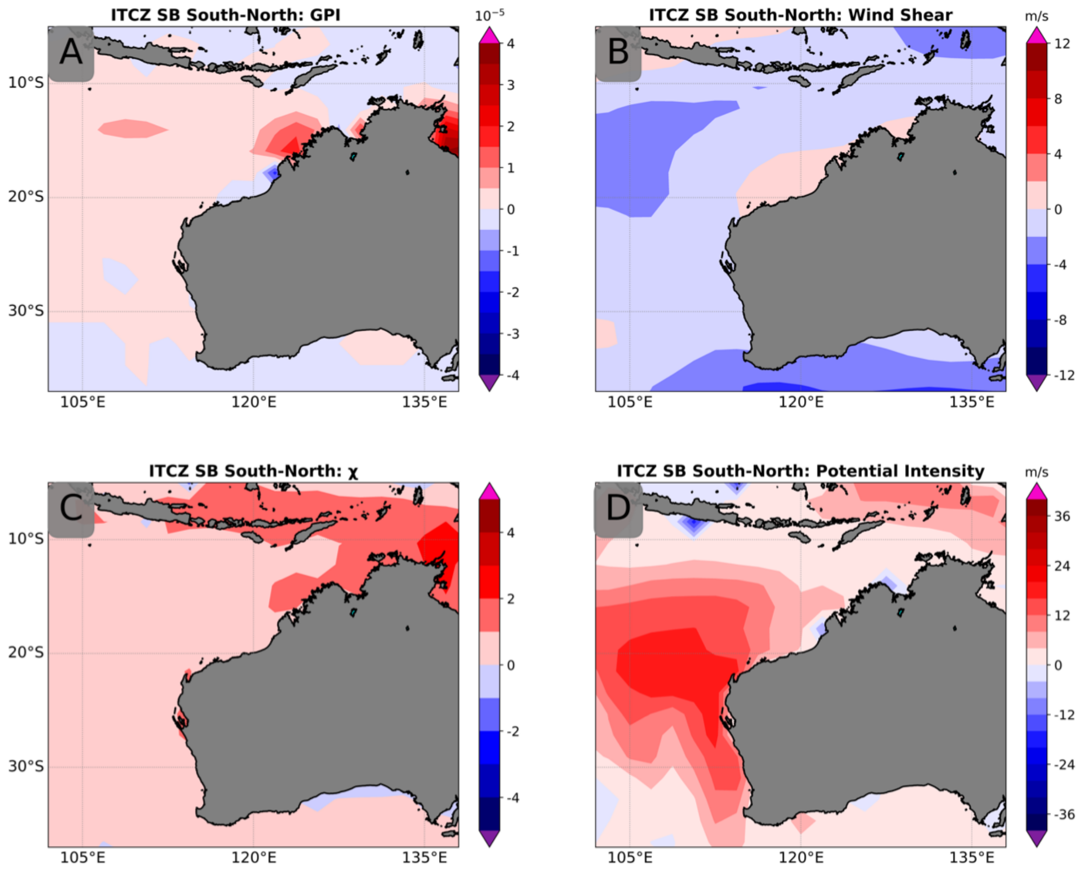

Figure S15. Genesis potential index (GPI) and associated controls on cyclogenesis. Same as Figure S14 but data for MPI-ESM.

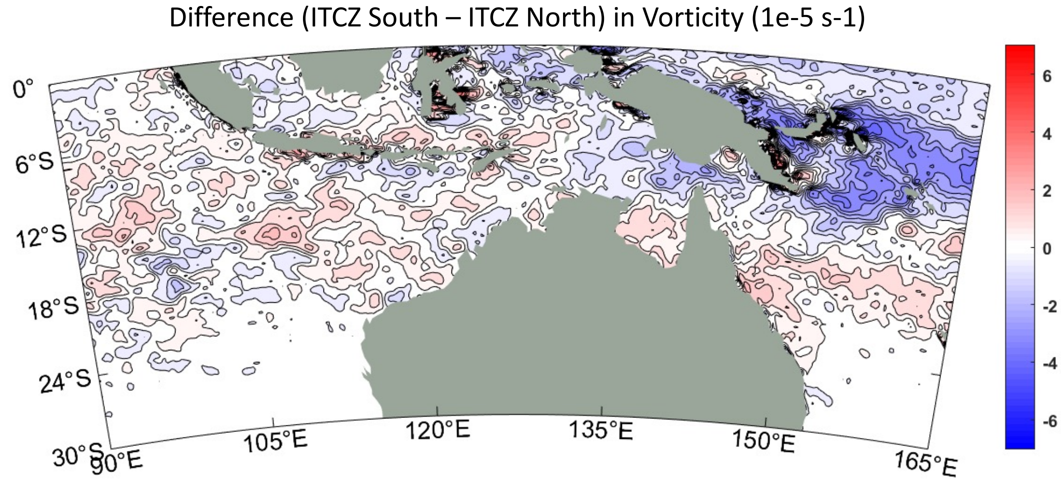

Figure S16. Differences in vorticity between southerly ITCZ and northerly ITCZ years. Data from the MPI-ESM for November - March. Unlike other parameters used to calculate the TC genesis potential index (middle tropospheric saturation deficit, vertical wind shear, and potential intensity), vorticity does not enhance cyclogenesis on the northwest shelf during a more southerly ITCZ.

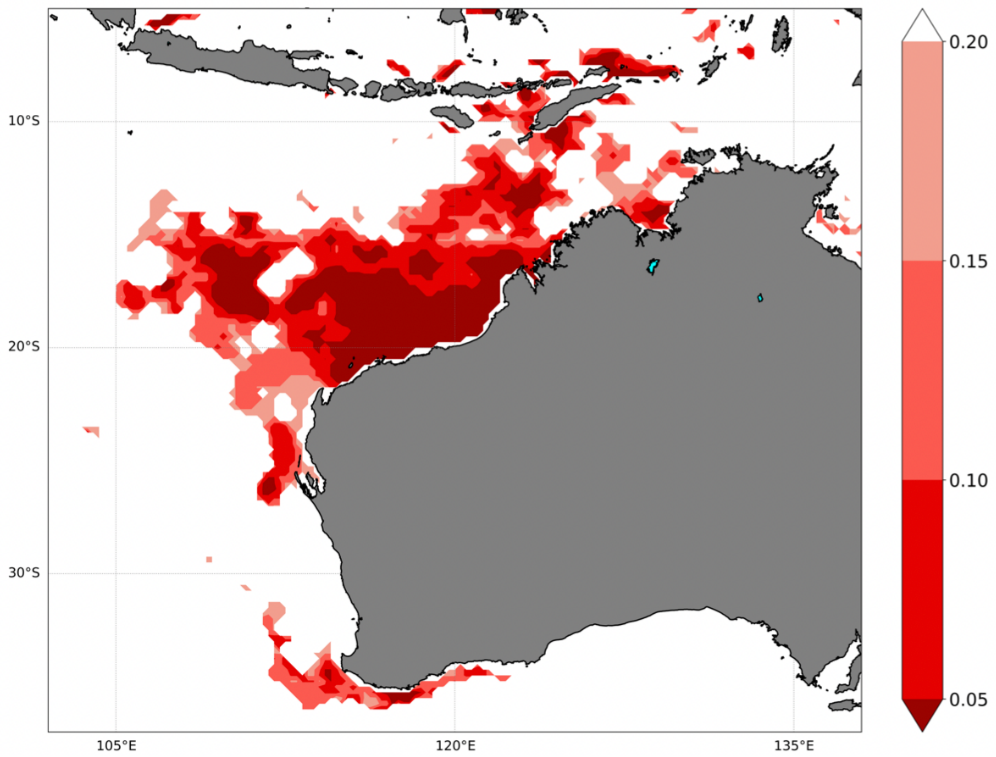

Figure S17. P-values for difference in GPI between southern vs northern ITCZ. Calculated using a Wilcoxon test using ERA5 data as shown in Figure 5.

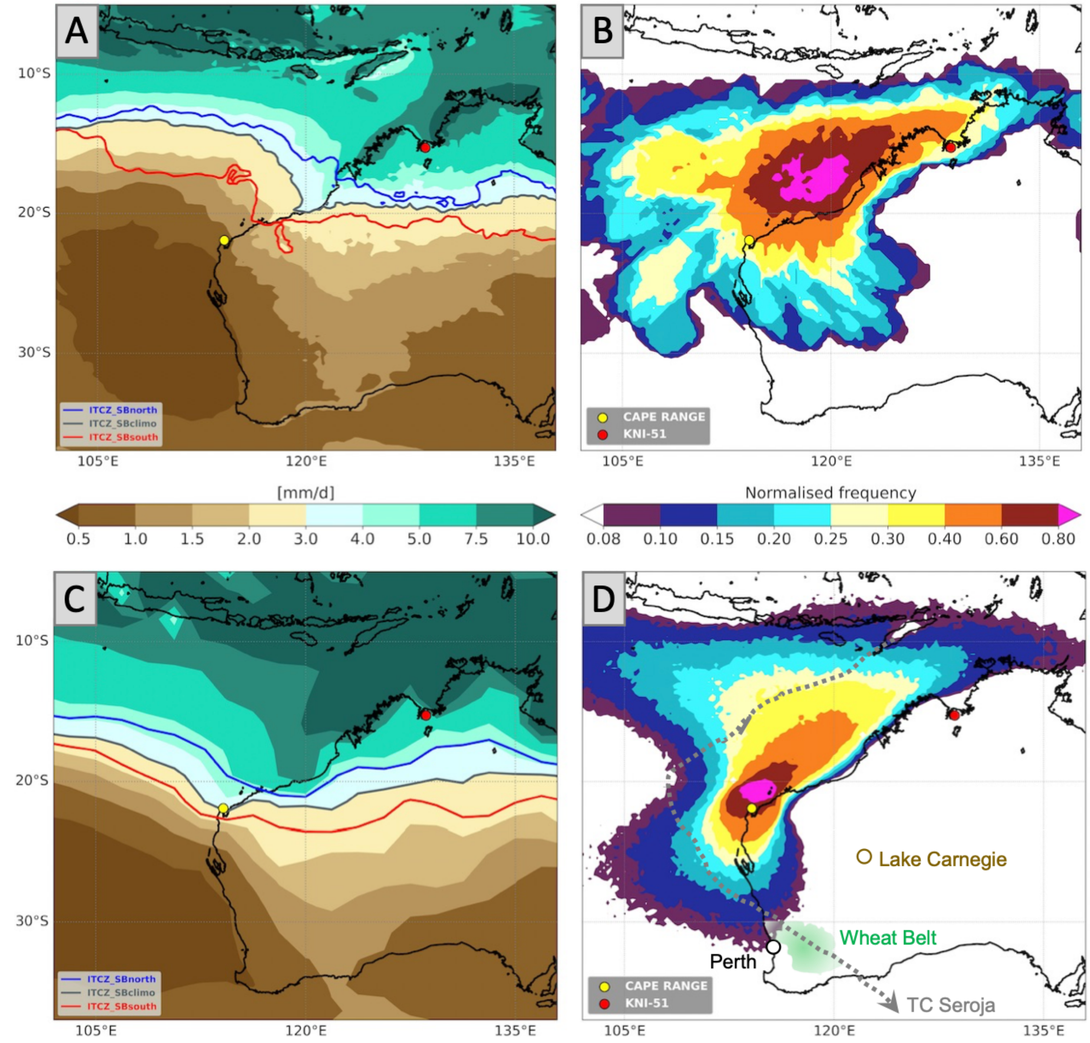

Figure S18. Australian tropical wet season and TC climatology from observations and model output. (A) Mean tropical wet season (December-March) rainfall calculated with ERA5 data for the period 1979-2018 for the Australian region with the southern boundary (SB) of the austral summer ITCZ (defined as the 3 mm/day isohyet) for the mean (grey), southward (red), northward (blue) positions (Methods). Circles denote locations of cave sites (KNI-51 = red; Cape Range = yellow). (B) Normalized TC frequency (number of TCs passing over each grid cell divided by the maximum value over the entire map) calculated with ERA5 data (spatial grid at 0.25° horizontal resolution) for the Australian region cyclone season (November-April) for CE 1979-2018 (Methods). (C) Same as (A) but for output from the Max Planck Institute Earth System Model "Past-1000 Year" (MPI-ESM) simulation. (D) Same as (B) but data from the MPI-ESM (Methods). Poleward shift in ITCZ southern boundary relative to the modern (see A) corresponds to southward migration of peak TC activity toward Cape Range shown in (D). Dotted line defines track of 2021 TC Seroja over the southwest Australia wheat belt (green shaded area). Lake Carnegie (brown circle), located in the desert interior, fills to overflowing in response to TC-derived rainfall in some years.

Table S1. U/Th Isotopic Ratios and Ages

Stalagmite	Distance to Top (mm)	^{238}U (ng/g)	^{232}Th (pg/g)	$\delta^{234}U$[a] (corr'd)	Error[b]	$^{230}Th/^{238}U$ (activity)	Error	$^{230}Th/^{232}Th$ (ppm)	Error	Uncorrected Year BP[c]	Error (yr)	Corrected Year BP[d]	Error (yr)
16-1	76.0	7,014	13,793	1,350.9	2.4	0.02957	0.00006	247.9	0.9	1,316	3	1,291	25
16-1	266	7,348	7,126	1,357.7	2.4	0.03175	0.00007	539.6	1.3	1,414	4	1402	13
16-1	382	6,682	16,339	1,341.3	2.3	0.03538	0.00026	238.6	1.9	1,597	12	1566	33
16-1	462	9,895	78,911	1,357.7	2.4	0.03967	0.00008	82.0	0.2	1,786	4	1688	99
16-2	46	5,078	13,966	1,261.0	2.3	0.02553	0.00006	153.0	0.4	1,173	3	1138	36
16-2	211	6,736	9,136	1,148.2	2.2	0.02575	0.00006	312.9	0.7	1,250	3	1231	19
16-2	335	5,364	11,717	1,251.0	2.3	0.02894	0.00008	218.5	3.1	1,347	4	1318	29
16-2	431	7,393	23,817	1,330.1	2.3	0.03283	0.00007	168.0	0.4	1,483	4	1443	40
16-4	39	5,487	4,901	1,204.6	6.0	0.01395	0.00003	257.4	0.7	626	2	614	12
16-4	147	6,905	724	1,129.1	2.1	0.01417	0.00008	2,228.0	226	663	4	661	4
16-4	178	5,417	7,300	1,095.8	2.1	0.01470	0.00004	179.8	0.5	702	2	683	19
16-4	185	5,623	306	1,098.7	2.1	0.01421	0.00009	4,302.0	1,019	675	5	673	5
16-4	196	6,140	557	1,118.5	2.1	0.01435	0.00008	2,608.0	198	676	4	674	4
16-4	215	5,653	16,969	1,066.0	2.1	0.01493	0.00005	82.0	0.4	725	3	683	42
16-4	361	6,812	12,719	1,181.5	2.2	0.01719	0.00004	151.7	0.4	797	2	772	25
16-6	19	6,185	9,520	1,138.7	2.1	0.01713	0.00004	182.4	0.4	806	2	785	21
16-6	184	2,064	3,791	1,034.1	2.0	0.01799	0.00005	161.6	0.6	903	3	877	26
16-6	328	6,992	16,095	1,157.3	2.2	0.02060	0.00005	147.5	0.4	981	3	950	31
16-7	33	11,395	15,224	1,237.1	2.2	0.00851	0.00002	104.9	0.3	349	1	332	17
16-7	92	6,115	6,738	1,071.5	2.1	0.00833	0.00005	124.7	1.1	374	2	358	16
16-7	112	4,206	40,734	1,172.9	2.2	0.01106	0.00006	18.8	0.1	491	3	361	130
16-7	172	4,793	17,706	1,072.4	2.1	0.01184	0.00004	52.8	0.2	559	2	507	52
16-7	267	6,675	12,116	1,197.2	2.2	0.01358	0.00003	123.3	0.3	610	2	586	24
16-7	348	3,932	46,400	1,333.8	2.4	0.0369	0.00012	51.6	0.2	1,675	6	1527	147

[a] $\delta^{234}U_{meas'd} = [(^{234}U/^{238}U)_{meas'd}/(^{234}U/^{238}U)_{eq}-1] \times 10^3$, where $(^{234}U/^{238}U)_{eq}$ is secular equilibrium activity ratio: $\lambda_{238}/\lambda_{234} = 1.0$. Values in permil.

[b] Errors are at the 2σ level.

[c] BP = before present where present is CE 1950.

[d] An initial $^{230}Th/^{232}Th$ atomic ratio of $4.4 \times 10^{-6} \pm 4.4 \times 10^{-6}$ was used to correct measured $^{230}Th/^{232}Th$ ratios for unsupported ^{230}Th.

Table S2. Comparison of ITCZ Position with Tropical Cyclones and Climate Modes

ITCZSB North[a]	*Rain (mm)*[b]	*TCs*[c]	*ENSO*[d]	*IOD*[e]	*SAM*[f]
1985-86	106	6	La Niña	Neutral	Neutral
1987-88	0	0	El Niño	Neutral	Neutral
1992-93	103	1	Neutral	Negative	Neutral
1993-94	129	4	Neutral	Neutral	Positive
1995-96	329	4[b]	La Niña	Neutral	Neutral
2001-02	0	0	Neutral	Neutral	Positive
2004-05	0	1	El Niño	Neutral	Neutral
TOTAL	*667*	*16*			
TC>10 mm		*8*			

ITCZSB South[a]	*Rain (mm)*	*TCs*	*ENSO*	*IOD*	*SAM*
1982-83	10	2	El Niño	Positive	Neutral
1990-91	30	3	El Niño	Neutral	Neutral
1996-97	27	4	La Niña	Negative	Neutral
1999-00	139	8	La Niña	Neutral	Neutral
2008-09	69	2	La Niña	Neutral	Neutral
2010-11	190	4	La Niña	Negative	Neutral
2011-12	48	3	La Niña	Neutral	Positive
TOTAL	*513*	*26*			
TC>10 mm		*9*			

[a] Years when the southern boundary (SB) of the austral summer ITCZ is more than 1 s.d. from the mean ITCZ position.
[b] Calculated for days when a TC is located within a Cape Range-centered box of 1° per side.
[c] Includes only TCs in 1995-96 with rainfall > 10 mm. During 1995-96, 241 mm of total 329 mm was due to TC Frank.
[d] El Niño-Southern Oscillation (ENSO) phases according NOAA Climate Prediction Center.
[e] Indian Ocean Dipole (IOD) phases according to Australian Bureau of Meteorology classification.
[f] Southern Annular Mode (SAM) phases according to Marshall Southern Annular Mode Index (*84*) using only summer (DJF) values, where positive (negative) categories are defined as falling outside 1 s.d. of the mean. Data from ref. 85.

Data S1. (separate file)

KNI-51 stalagmite oxygen isotopes and model ages, Cape Range stalagmite oxygen isotope data and laminae ages, rainwater amounts and oxygen isotope ratios, and TC downscaling data.

REFERENCES AND NOTES

1. P. Peduzzi, B. Chatenoux, H. Dao, A. De Bono, C. Herold, J. Kossin, F. Mouton, O. Nordbeck, Global trends in tropical cyclone risk. *Nat. Clim. Change* **2**, 289–294 (2012).

2. G. S. McGrath, R. Sadler, K. Fleming, P. Tregoning, C. Hinz, E. J. Veneklaas, Tropical cyclones and the ecohydrology of Australia's recent continental-scale drought. *Geophys. Res. Lett.* **39**, L03404 (2012).

3. M. F. Lewis, G. R. Walker, Assessing the potential for significant and episodic recharge in southwestern Australia using rainfall data. *Hydrogeo. J.* **10**, 229–237 (2002).

4. J. Kossin, K. Emanuel, G. Vecchi, The poleward migration of the location of tropical cyclone maximum intensity. *Nature* **509**, 349–352 (2014).

5. S. Sharmila, H. Hendon, Mechanisms of multiyear variations of Northern Australia wet-season rainfall. *Sci. Rep.* **10**, 5086 (2020).

6. T. M. Merlis, M. Zhao, I. M. Held, The sensitivity of hurricane frequency to ITCZ changes and radiatively forced warming in aquaplanet simulations. *Geophys. Res. Lett.* **40**, 4109–4114 (2013).

7. G. Berry, M. J. Reeder, Objective identification of the intertropical convergence zone: Climatology and trends from the ERA-Interim. *J. Climate* **27**, 1894–1909 (2014).

8. T. Schneider, T. Bischoff, G. Haug, Migrations and dynamics of the intertropical convergence zone. *Nature* **513**, 45–53 (2014).

9. R. F. Denniston, C. C. Ummenhofer, A. D. Wanamaker, M. S. Lachniet, G. Villarini, Y. Asmerom, V. J. Polyak, K. J. Passaro, J. Cugley, D. Woods, W. F. Humphreys, Expansion and contraction of the Indo-Pacific tropical rain belt over the last three millennia. *Sci. Rep.* **6**, 34485 (2016).

10. S. Evans, E. Dawson, P. Ginoux, Linear relation between shifting ITCZ and dust hemispheric asymmetry. *Geophys. Res. Lett.* **47**, e2020GL090499 (2020).

11. H. E. Ridley, Y. Asmerom, J. U. L. Baldini, S. F. M. Breitenbach, V. V. Aquino, K. M. Prufer, B. J. Culleton, V. Polyak, F. A. Lechleitner, D. J. Kennett, M. Zhang, N. Marwan, C. G. Macpherson, L. M.

Baldini, T. Xiao, J. L. Peterkin, J. Awe, G. H. Haug, Aerosol forcing of the position of the intertropical convergence zone since AD 1550. *Nat. Geosci.* **8**, 195–200 (2015).

12. Y. Wang, H. Cheng, R. L. Edwards, Y. He, X. Kong, Z. An, J. Wu, M. J. Kelly, C. A. Dykoski, X. Li, The Holocene Asian monsoon: Links to solar changes and North Atlantic climate. *Science* **308**, 854–857 (2005).

13. J. C. H. Chiang, C. M. Bitz, Influence of high latitude ice cover on the marine intertropical convergence zone. *Clim. Dyn.* **25**, 477–496 (2005).

14. M. E. Mann, Z. Zhang, S. Rutherford, R. S. Bradley, M. K. Hughes, D. Shindell, C. Ammann, G. Faluvegi, F. Ni, Global signatures and dynamical origins of the little ice age and medieval climate anomaly. *Science* **326**, 1256–1260 (2009).

15. P. Maher, S. Sherwood, Skill in simulating Australian precipitation at the tropical edge. *J. Climate* **29**, 1477–1496 (2016).

16. S. J. Camargo, Global and regional aspects of tropical cyclone activity in the CMIP5 models. *J. Climate* **26**, 9880–9902 (2013).

17. T. A. McCloskey, J. T. Knowles, Migration of the tropical cyclone zone throughout the Holocene, in *Hurricanes and Climate Change*, B. J. Elsner, H. T. Jagger, Eds. (Springer U.S, 2009) pp. 169–187).

18. J. Altman, O. N. Ukhvatkina, A. M. Omelko, M. Macek, T. Plener, V. Pejcha, T. Cerny, P. Petrik, M. Srutek, J-S. Song, A. A. Zhmereknetsky, A. S. Vozmischeva, P. V. Krestov, T. Y. Petrenko, K. Treydte, J. Dolezal, Poleward migration of the destructive effects of tropical cyclones during the 20th century. *Proc. Nat. Acad. Sci. U.S.A.* **115**, 11543–11548 (2018).

19. L. M. Baldini, J. U. L. Baldini, J. N. McElwaine, A. B. Frappier, Y. Asmerom, K.-B. Liu, K. M. Prufer, H. E. Ridley, V. Polyak, D. J. Kennett, C. G. Macpherson, V. V. Aquino, J. Awe, S. F. M. Breitenbach, Persistent northward North Atlantic tropical cyclone track migration over the past five centuries. *Sci. Rep.* **6**, 37522 (2016).

20. J. F. Bramante, M. R. Ford, P. S. Kench, A. A. Ashton, M. R. Toomey, R. M. Sullivan, K. B. Karnauskas, C. C. Ummenhofer, J. P. Donnelly, Increased typhoon activity in the Pacific deep tropics driven by Little Ice Age circulation changes. *Nat. Geosci.* **13**, 806–811 (2020).

21. H.-F. Chen, Y.-C. Liu, C.-W. Chiang, X. Liu, Y.-M. Chou, H.-J. Pan, China's historical record when searching for tropical cyclones corresponding to intertropical convergence zone (ITCZ) shifts over the past 2 kyr. *Clim. Past* **15**, 279–289 (2019).

22. P. J. van Hengstum, J. P. Donnelly, P. L. Fall, M. R. Toomey, N. A. Albury, B. Kakuk, The intertropical convergence zone modulates intense hurricane strikes on the western North Atlantic margin. *Sci. Rep.* **6**, 21728 (2016).

23. J. Haig, J. Nott, G.-J. Reichart, Australian tropical cyclone activity lower than at any time over the past 550-1,500 years. *Nature* **505**, 667–671 (2014).

24. R. A. Dare, N. E. Davidson, J. L. McBride, Tropical cyclone contribution to rainfall over Australia. *Mon. Weath. Rev.* **140**, 3606–3619 (2012).

25. B. Ng, K. Walsh, S. Lavender, The contribution of tropical cyclones to rainfall in northwest Australia. *Inter. J. Clim.* **35**, 2689–2697 (2014).

26. W. Dansgaard, Stable isotopes in precipitation. *Tellus* **16**, 436–468 (1964).

27. C. Zwart, N. C. Munksgaard, N. Kurita, M. J. Bird, Stable isotopic signature of Australian monsoon controlled by regional convection. *Quat. Sci. Rev.* **151**, 228–235 (2016).

28. C. Risi, S. Bony, F. Vimeux, Influence of convective processes on the isotopic composition ($\delta^{18}O$ and δD) of precipitation and water vapor in the tropics: 2. Physical interpretation of the amount effect. *J. Geophys. Res.* **113**, D19306 (2008).

29. C. Sun, L. Tian, T. M. Shanahan, J. W. Partin, Y. Gao, N. Piatrunia, J. Banner, Isotopic variability in tropical cyclone precipitation is controlled by Rayleigh distillation and cloud microphysics. *Comm. Earth Env.* **3**, 50 (2022).

30. R. F. Denniston, K.-H. Wyrwoll, V. Polyak, J. Brown, Y. Asmerom, A. D. Wanamaker Jr., Z. LaPointe, R. Ellerbroek, M. Barthelmes, D. Cleary, J. Cugley, D. Woods, W. F. Humphreys, A stalagmite record of Holocene Indonesian-Australian summer monsoon variability from the Australian tropics. *Quat. Sci. Rev.* **78**, 155–168 (2013).

31. M. S. Lachniet, Climatic and environmental controls on speleothem oxygen isotope values. *Quat. Sci. Rev.* **28**, 412–432 (2009).

32. M. S. Lachniet, Are aragonite stalagmites reliable paleoclimate proxies? Tests for oxygen isotope time-series replication and equilibrium. *Geol. Soc. Amer. Bull.* **127**, 1521–1533 (2015).

33. N. C. Munksgaard, C. Zwart, N. Kurita, A. Bass, J. Nott, M. J. Bird, Stable isotope anatomy of tropical cyclone Ita, north-eastern Australia, April 2014. *PLOS ONE* **10**, e0119728 (2015).

34. S. L. Lavender, A. J. Dowdy, Tropical cyclone track direction climatology and its intraseasonal variability in the Australian region. *J. Geophys. Res. Atm.* **121**, 13,236–13,249 (2016).

35. G. Villarini, R. F. Denniston, Contribution of tropical cyclones to extreme rainfall in Australia. *Inter. J. Clim.* **36**, 1019–1025 (2016).

36. K. J. Reid, I. Simmonds, C. L. Vincent, A. D. King, The Australian northwest cloudband: Climatology, mechanisms, and association with precipitation. *J. Climate* **32**, 6665–6684 (2019).

37. R. F. Denniston, G. Villarini, A. N. Gonzales, K.-H. Wyrwoll, V. J. Polyak, C. C. Ummenhofer, M. S. Lachniet, A. D. Wanamaker Jr., W. F. Humphreys, D. Woods, J. Cugley, Extreme rainfall activity in the Australian tropics reflects changes in the El Niño/Southern Oscillation over the last two millennia. *Proc. Nat. Acad. Sci. U.S.A.* **112**, 4576–4581 (2015).

38. C.-C. Shen, K. Lin, W. Duan, X. Jiang, J. W. Partin, R. L. Edwards, H. Cheng, M. Tan, Testing the annual nature of speleothem banding. *Sci. Rep.* **3**, 2633 (2013).

39. A. Rouillard, G. Skrzypek, C. Turney, S. Dogramci, Q. Hua, A. Zawadzki, J. Reeves, P. Greenwood, A. J. O'Donnell, P. F. Grierson, Evidence for extreme floods in arid subtropical northwest Australia during the Little Ice Age chronozone (CE 1400-1850). *Quat. Sci. Rev.* **144**, 107–122 (2016).

40. H. Hersbach, B. Bell, P. Berrisford, S. Hirahara, A. Horanyi, J. MuñozSabater, J. Nicolas, C. Peubey, R. Radu, D. Schepers, A. Simmons, C. Soci, S. Abdalla, X. Abellan, G. Balsamo, P. Bechtold, G. Biavati, J. Bidlot, M. Bonavita, G. De Chiara, P. Dahlgren, D. Dee, M. Diamantakis, R. Dragani, J. Flemming, R. Forbes, M. Fuentes, A. Geer, L. Haimberger, S. Healy, R. J. Hogan, E. Hólm, M. Janisková, S. Keeley, P. Laloyaux, P. Lopez, C. Lupu, G. Radnoti, P. de Rosnay, I. Rozum, F. Vamborg, S. Villaume, J-N. Thépaut, The ERA5 global reanalysis. *Quar. J. Royal Met. Soc.* **146**, 1999–2049 (2020).

41. K. R. Knapp, M. C. Kruk, D. H. Levinson, H. J. Diamon, C. J. Neumann, The International best track archive for climate stewardship (IBTrACS). *Bull. Amer. Met. Soc.* **91**, 363–376 (2010).

42. K. A. Emanuel, S. Ravela, E. Vivant, C. Risi, A statistical-deterministic approach to hurricane risk assessment. *Bull. Amer. Met. Soc* **19**, 299–314 (2006).

43. K. Emanuel, R. Sundararajan, J. Williams, Hurricanes and global warming: Results from downscaling IPCC AR4 simulations. *Bull. Amer. Meteor. Soc.* **89**, 347–368 (2008).

44. A. D. Magee, A. S. Kiem, Using indicators of ENSO, IOD, and SAM to improve lead time and accuracy of tropical cyclone outlooks for Australia. *J. App. Meteor. Clim.* **59**, 1901–1917 (2020).

45. J. S. Wijnands, G. Qian, K. L. Shelton, R. J. B. Fawcett, J. C. L. Chan, Y. Kuleshov, Seasonal forecasting of tropical cyclone activity in the Australian and the South Pacific Ocean regions. *Math. Clim. Weath. Forecast.* **1**, 21–42 (2015).

46. S. S. Chand, J. L. McBride, K. J. Tory, M. C. Wheeler, Impact of different ENSO regimes on southwest Pacific tropical cyclones. *J. Climate* **26**, 600–608 (2013).

47. H. A. Ramsay, L. M. Leslie, P. J. Lamb, M. B. Richman, M. Leplastrier, Interannual variability of tropical cyclones in the Australian region: Role of large-scale environment. *J. Climate* **21**, 1083–1103 (2008).

48. K. H. Goebbert, L. M. Leslie, Interannual variability of northwest Australian tropical cyclone. *J. Climate* **23**, 4538–4555 (2010).

49. Z. Lu, Z. Liu, J. Zhu, K. M. Cobb, A review of paleo El Niño-Southern Oscillation. *Atmos.* **9**, 130 (2018).

50. Intergovernmental Panel on Climate Change (IPCC), Summary for policymakers, in *Climate Change 2021: The Physical Science Basis. Contribution of Working Group I to the Sixth Assessment Report of the Intergovernmental Panel on Climate Change*, V. Masson-Delmotte, P. Zhai, A. Pirani, S. L. Connors, C. PÉan, S. Berger, N. Caud, Y. Chen, L. Goldfarb, M. I. Gomis, M. Huang, K. Leitzell, E. Lonnoy, J. B. R. Matthews, T. K. Maycock, T. Waterfield, O. Yelekçi, R. Yu, B. Zhou, Eds. (Cambridge Univ. Press, 2021).

51. J. H. Jungclaus, N. Fischer, H. Haak, K. Lohmann, J. Marotzke, D. Matel, U. Mikolajewics, D. Notz, J. A. von Storch, Characteristics of the ocean simulations in the Max Planck Institute Ocean Model (MPIOM) the ocean component of the MPI-Earth system model. *J. Adv. Model. Earth Syst.* **5**, 422–446 (2013).

52. K. Emanuel, Increasing destructiveness of tropical cyclones over the past 30 years. *Nature* **436**, 686–688 (2005).

53. W. Cai, T. Cowan, M. Thatcher, Rainfall reductions over Southern Hemisphere semi-arid regions: The role of subtropical dry zone expansion. *Sci. Rep.* **2**, 702 (2012).

54. M. Previdi, B. G. Liepert, Annular modes and Hadley cell expansion under global warming. *Geophys. Res. Lett.* **34**, L22701 (2007).

55. D. J. Seidel, Q. Fu, J. Randel, T. J. Reichler, Widening of the tropical belt in a changing climate. *Nat. Geosci.* **1**, 21–24 (2008).

56. J. L. McCallum, R. S. Crosbie, G. R. Walker, W. R. Dawes, Impacts of climate change on groundwater in Australia: A sensitivity analysis of recharge. *Hydrogeo. J.* **18**, 1625–1638 (2010).

57. R. Sudmeyer, A. Edward, V. Fazakerley, L. Simpkin, I. Foster, *Climate Change: Impacts and Adaptation for Agriculture in Western Australia* (Bulletin 4870, Department of Agriculture and Food, West Australia, Perth, 2016).

58. S. S. Bell, S. S. Chand, K. J. Tory, A. J. Dowdy, C. Turville, H. Ye, Projections of southern hemisphere tropical cyclone track density using CMIP5 models. *Clim. Dyn.* **52**, 6065–6079 (2019).

59. J. Studholme, A. V. Fedorov, S. K. Gulev, K. Emanuel, K. Hodges, Poleward expansion of tropical cyclone latitudes in warming climates. *Nat. Geosci.* **15**, 14–28 (2022).

60. A. Mamalakis, J. T. Randerson, J.-Y. Yu, M. S. Pritchard, G. Magnusdottir, P. Smyth, P. A. Levine, S. Yu, E. Foufoula-Georgiou, Zonally contrasting shifts of the tropical rain belt in response to climate change. *Nat. Clim. Change* **11**, 143–151 (2021).

61. M. P. Byrne, A. G. Pendergrass, A. D. Rapp, K. R. Wodzicki, Response of the intertropical convergence zone to climate change: Location, width, and strength. *Curr. Clim. Change Rep.* **4**, 355–370 (2018).

62. E. Scoccimarro, S. Gualdi, G. Villarini, G. A. Vecchi, M. Zhao, K. Walsh, A. Naavarra, Intense precipitation events associated with landfalling tropical cyclones in response to a warmer climate and increased CO_2. *J. Climate* **27**, 4642–4654 (2014).

63. W. Duan, J. Ruan, W. Luo, T. Li, L. Tian, G. Zeng, D. Zhang, Y. Bai, J. Li, T. Tao, P. Zhang, A. Baker, M. Tan, The transfer of seasonal isotopic variability between precipitation and drip water at eight caves in the monsoon regions of China. *Geochim. Cosmochim. Acta* **183**, 250–266 (2016).

64. M. Markowska, M. O. Cuthbert, A. Baker, P. C. Treble, M. S. Anderson, L. Adler, A. Griffiths, S. Frisia, Modern speleothem oxygen isotope hydroclimate records in water-limited SE Australia. *Geochim. Cosmochim. Acta* **270**, 431–448 (2020).

65. S. Frisia, A. Borsato, J. Hellstrom, High spatial resolution investigation of nucleation, growth and early diagenesis in speleothems as exemplar for sedimentary carbonates. *Ear.-Sci. Rev.* **178**, 68–91 (2018).

66. R. Martín-García, A. M. Alonsa-Zarza, S. Frisia, A. Rodríguez-Berriguete, R. Drysdale, J. Hellstrom, Effect of aragonite to calcite transformation on the geochemistry and dating accuracy of speleothems. An example of Castañar Cave, Spain. *Sediment.* **383**, 41–54 (2019).

67. J. A. Dorale, Z. Liu, Limitations of Hendy Test criteria in judging the paleoclimatic suitability of speleothems and the need for replication. *J. Cave Karst Stud.*, **71**, 73–80 (2009).

68. M. L. Griffiths, R. N. Drysdale, M. K. Gagan, J.-x. Zhao, L. K. Ayliffe, J. C. Hellstrom, W. S. Hantoro, S. Frisia, Y.-x. Feng, I. Cartwright, E. St. Pierre, M. J. Fischer, B. W. Suwargadi, Increasing Australian-Indonesian monsoon rainfall linked to early Holocene sea-level rise. *Nat. Geosci.* **2**, 636–639 (2009).

69. S. F. M. Breitenbach, K. Rehfeld, B. Goswami, J. U. L. Baldini, H. E. Ridley, D. J. Kennett, K. M. Prufer, V. V. Aquino, Y. Asmerom, V. J. Polyak, H. Cheng, J. Kurths, N. Marwan, COnstructing proxy records from age models (COPRA). *Clim. Past* **8**, 1765–1779 (2012).

70. E. Weller, W. Cai, S.-K. Min, L. Wu, K. Ashok, T. Yamagata, More frequent extreme northward shifts of eastern Indian Ocean tropical convergence under greenhouse warming. *Sci. Rep.* **4**, 6087 (2014).

71. A. C. Davison, D. V. Hinckley, *Bootstrap Methods and Their Application* (Cambridge Series on Statistical and Probabilistic Mathematics, Cambridge Univ. Press, 1997).

72. S. Dandoy, F. S. R. Pausata, S. J. Camargo, R. Laprise, K. Winger, K. Emanuel, Atlantic hurricane response to Saharan greening and reduced dust emissions during the mid-Holocene. *Clim. Past* **17**, 675–701 (2021).

73. E. Scoccimarro, S. Gualdi, A. Bellucci, A. Sanna, P. G. Pogli, E. Mazini, M. Vichi, P. Oddo, A. Navarra, Effects of tropical cyclones on ocean heat transport in a high-resolution coupled general circulation model. *J. Climate* **24**, 4368–4384 (2011).

74. K. Walsh, M. Fiorino, C. W. Landsea, K. L. McInnes, Objectively determined resolution-dependent threshold criteria for the detection of tropical cyclones in climate models and reanalyses. *J. Climate* **20**, 2307–2314 (2007).

75. L.-P. Caron, C. G. Jones, P. A. Vaillancourt, K. Winger, On the relationship between cloud-radiation interaction, atmospheric stability and Atlantic tropical cyclones in a variable-resolution climate model. *Clim. Dyn.* **40**, 1257–1269 (2012).

76. R. J. van der Ent, L. Wang-Erlandsson, P. W. Keys, H. H. G. Savenije, Contrasting roles of interception and transpiration in the hydrological cycle—Part 2: Moisture recycling. *Ear. Syst. Dyn.* **5**, 471–489 (2014).

77. K. Emanuel, E. Rappaport, Forecast skill of a simplified hurricane intensity prediction model, in *Proceedings of the 24th Conference on Hurricanes and Tropical Meteorology*, Ft. Lauderdale, FL, Amer. Meteor. Soc., CD-ROM, 6A.5 (2000).

78. S.-P. Xie, The Hadley circulation: Present, past and future, in *Advances in Global Change Research*, H. F. Diaz, R. S. Bradley, Eds. (Kluwer Academic, 2004), vol. 21, pp. 121–152.

79. A. C. Burnett, A. Sheshadri, L. G. Silvers, T. Robinson, Tropical cyclone frequency under varying SSTs in aquaplanet simulations. *Geophys. Res. Lett.* **48**, e2020GL091980 (2021).

80. F. S. R. Pausata, S. J. Camargo, Tropical cyclone activity affected by volcanically induced ITCZ shifts. *Proc. Nat. Acad. Sci. U.S.A.* **116**, 7732–7737 (2019).

81. Australian Bureau of Meteorology, "Australian Gridded Climate Data (AGCD); v2.0.0 Snapshot (1900-01-01 to 2020-05-31)" (2020).

82. A. Martín-García, A. M. Alonso-Zarza, S. Frisia, Á. Rodríguez-Berriguete, R. Drysdale, J. Hellstrom, Effect of aragonite to calcite transformation on the geochemistry and dating accuracy of speleothems. An example from Castañar Cave, Spain. *Sedimentary Geology* **383**, 41–54 (2019).

83. S. J. Camargo, A. H. Sobel, Western North Pacific tropical cyclone intensity and ENSO. *J. Climate* **18**, 2996–3006 (2005).

84. G. J. Marshall, Trends in the Southern annular mode from observations and reanalyses. *J. Climate* **16**, 4134–4143 (2003).

85. G. Marshall, "The Climate Data Guide: Marshall Southern Annular Mode (SAM) Index (Station-based)" (National Center for Atmospheric Research Staff, Eds). Last modified 19 March 2018. https://climatedataguide.ucar.edu/climate-data/marshall-southern-annular-mode-sam-index-station-based.
